# Usher syndrome proteins ADGRV1 (USH2C) and CIB2 (USH1J) interact and share a common interactome containing TRiC/CCT-BBS chaperonins

**DOI:** 10.3389/fcell.2023.1199069

**Published:** 2023-06-22

**Authors:** Joshua Linnert, Barbara Knapp, Baran E. Güler, Karsten Boldt, Marius Ueffing, Uwe Wolfrum

**Affiliations:** ^1^ Institute of Molecular Physiology, Molecular Cell Biology, Johannes Gutenberg University Mainz, Mainz, Germany; ^2^ Institute for Ophthalmic Research, Eberhard Karls University of Tuebingen, Tubingen, Germany

**Keywords:** usher syndrome, bardet biedl syndrome (BBS), protein networks, retinal ciliopathies, VLGR1, TRiC/CCT chaperonins, primary cilia, photoreceptor cells

## Abstract

The human Usher syndrome (USH) is the most common form of a sensory hereditary ciliopathy characterized by progressive vision and hearing loss. Mutations in the genes *ADGRV1* and *CIB2* have been associated with two distinct sub-types of USH, namely, USH2C and USH1J. The proteins encoded by the two genes belong to very distinct protein families: the adhesion G protein-coupled receptor ADGRV1 also known as the very large G protein-coupled receptor 1 (VLGR1) and the Ca^2+^- and integrin-binding protein 2 (CIB2), respectively. In the absence of tangible knowledge of the molecular function of ADGRV1 and CIB2, pathomechanisms underlying USH2C and USH1J are still unknown. Here, we aimed to enlighten the cellular functions of CIB2 and ADGRV1 by the identification of interacting proteins, a knowledge that is commonly indicative of cellular functions. Applying affinity proteomics by tandem affinity purification in combination with mass spectrometry, we identified novel potential binding partners of the CIB2 protein and compared these with the data set we previously obtained for ADGRV1. Surprisingly, the interactomes of both USH proteins showed a high degree of overlap indicating their integration in common networks, cellular pathways and functional modules which we confirmed by GO term analysis. Validation of protein interactions revealed that ADGRV1 and CIB2 mutually interact. In addition, we showed that the USH proteins also interact with the TRiC/CCT chaperonin complex and the Bardet Biedl syndrome (BBS) chaperonin-like proteins. Immunohistochemistry on retinal sections demonstrated the co-localization of the interacting partners at the photoreceptor cilia, supporting the role of USH proteins ADGRV1 and CIB2 in primary cilia function. The interconnection of protein networks involved in the pathogenesis of both syndromic retinal dystrophies BBS and USH suggest shared pathomechanisms for both syndromes on the molecular level.

## Introduction

The human Usher syndrome (USH) is a clinically and genetically heterogeneous autosomal recessive disorder characterized by deafness and vestibular dysfunction combined with vision loss due to *Retinitis pigmentosa* (Reiners et al., 2006; Delmaghani and El-Amraoui, 2022). Three types of USH (USH1, USH2 and USH3) are distinguished, based on the age of onset, disease progression and the severity of the symptoms. To date, only one gene for USH3, *CLRN1*, three genes for USH2, *USH2A*, *ADGRV1* (USH2C), *WHRN* (USH2D), and six USH1 genes, *MYO7A* (USH1B), *USH1C, CDH23* (USH1D), *PCDH15* (USH1F), *USH1G,* and *CIB2* (USH1J) have been assigned to USH (Fuster-García et al., 2021). Recently, the association of mutations in CIB2 with USH and the assignment to USH1J (Riazuddin et al., 2012) has been debated (Dal Cortivo and Dell’orco, 2022; Delmaghani and El-Amraoui, 2022). Clinical analysis on patients with confirmed mutations in *CIB2*, a NGS meta-analysis of USH patients, and work on *cib2* mouse models have recently raised doubts that *CIB2* is a USH-causing gene, but rather a gene for non-syndromic deafness (DFNB48) (Michel et al., 2017; Booth et al., 2018; Jouret et al., 2019). However, a recent study found a distinct visual phenotype alongside deafness in a *cib2*-deficient mouse model, confirming the association of *CIB2* defects with syndromic inherited retinal dystrophies (IRD) such as USH (Sethna et al., 2021).

The various USH genes encode very heterogeneous families and groups of proteins, such as scaffold proteins, transmembrane proteins, or motor proteins, but they share the common feature of being involved in common protein networks called the USH interactome (Reiners et al., 2006; Mathur and Yang, 2015). However, the cellular function of these USH proteins in photoreceptors and hair cells has not been fully elucidated, an understanding that would be necessary to mitigate the phenotypic burden of mutations in any of the USH genes by means of sound treatment. Here, we focus on *ADGRV1* (USH2C) and *CIB2* (USH1J) that codify the very large G protein-coupled receptor 1 (VLGR1) ADGRV1 and the Ca^2+^- and integrin-binding protein 2 (CIB2), respectively.

**FIGURE 1:**
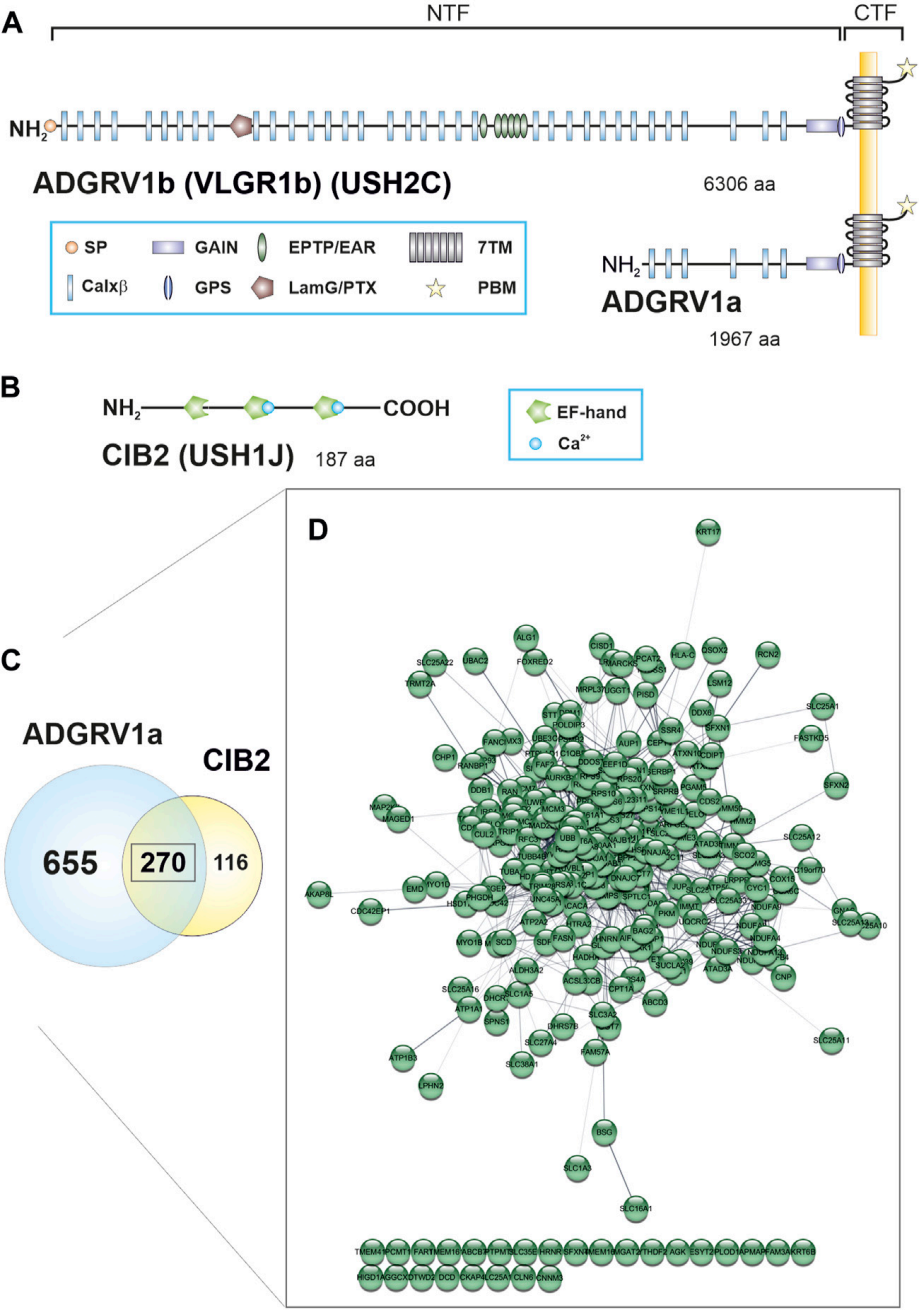
ADGRV1 and CIB2 share a common protein network. **(A)** The human full-length ADGRV1 isoform b contains 6,306 amino acids (aa), the ADGRV1a isoform 1,967 aa. Both isoforms can undergo autocleavage at the G protein-coupled receptor proteolytic cleavage site (GPS), resulting in an N-terminal fragment (NTF) and a C-terminal fragment (CTF). The NTF of ADGRV1b contains a signal peptide (SP), 35 calcium-binding Calxβ domains, a laminin G/pentraxin domain (LamG/PTX), epitempin/epilepsy-associated repeats (EPTP/EAR) and the N-terminal part of the G protein-coupled receptor (GPCR) autoproteolysis-inducing (GAIN) domain. The CTF contains the seven transmembrane (7TM) domain and the C-terminal PDZ binding motif (PBM). **(B)** The CIB2 isoform 1 is composed of 187 aa and contains three EF-hand motifs. Only the last two motifs can bind calcium. **(C)** 925 binding partners were identified for ADGRV1a and 386 interactors were found for CIB2 by TAP analysis. 270 prey proteins were contained in both data sets. **(D)** Visualization of the common ADGRV1 and CIB2 network with the STRING application in Cytoscape (confidence view). Most prey (244 out of 270) show a high degree of connectivity, based on STRING interaction data.

CIB2 shares sequence identity with calmodulin and calcineurin B and contains three EF-hand domains, whereby only the last two domains can bind Ca^2+^ (Figure 1) (Dal Cortivo and Dell’orco, 2022). CIB2 is expressed in diverse tissues and cell types, such as the skeletal muscle, platelet cells, diverse nervous tissue as well as the sensory cells in the retina and the inner ear (Riazuddin et al., 2012; Jacoszek et al., 2017). CIB2 is involved in the regulation of Ca^2+^-homeostasis and interacts with integrins (Häger et al., 2008), important for hair cell differentiation and stereocilia development (Evans and Müller, 2000). In the eye, CIB2 is found in the neuronal retina and the retinal pigment epithelium where it participates in mTORC1 signaling and autophagy (Sethna et al., 2021).

The ADGRV1 protein, also known as VLGR1, GPR98, MASS1, or FEB4 is a seven-transmembrane receptor that belongs to the adhesion GPCR (ADGR) family (McMillan and White, 2010; Hamann et al., 2015). The characteristic very long extracellular domain of ADGRV1 comprises 35 Ca^2+^-binding Calx-beta domains (Calxβ), a laminin G/pentraxin domain (LamG/PTX), six epitempin/epilepsy-associated repeats (EPTP/EAR) and a G protein-coupled receptor proteolytic site (GPS), which is embedded in the GPCR autoproteolysis-inducing (GAIN) domain and divides the molecule in a N-terminal fragment (NTF) and a C-terminal fragment (CTF) (Figure 1A). The very C-terminal intracellular domain of ADGRV1 displays a terminal class I PDZ-binding motif (PBM).

Like CIB2, ADGRV1 is expressed in various tissues, most abundantly in the retina, the inner ear and the brain (Reiners et al., 2006; McMillan and White, 2010). ADGRV1 is essential for the formation of ankle-links during the development of hair cells (McGee et al., 2006; Michalski et al., 2007; Yagi et al., 2007). Defects in *Adgrv1* result in disorganized hair bundles, which manifest in hearing impairment. In photoreceptor cells, ADGRV1 builds fibrous links between the apical inner segment and the connecting cilium, which resemble the ankle links in hair cells and are lost in *Adgrv1* mutant mice (Maerker et al., 2008).

In the past years, it became evident that USH proteins are part of larger protein networks that are present in cilia, antenna-like structures that emerge from the cell surface (Van Wijk et al., 2009; Wright et al., 2012; May-Simera et al., 2017; Sorusch et al., 2017). Diseases affecting ciliary function - so-called ciliopathies - include besides USH, for example, also the Bardet Biedl syndrome (BBS) and numerous non-syndromic IRDs such as Leber congenital amaurosis (LCA) (Bettencourt-Dias et al., 2011; Braun and Hildebrandt, 2017; Bujakowska et al., 2017). The molecular mechanisms underlying the pathogenesis of these diseases are largely unknown and their analysis is challenging.

In recent studies, we have identified novel functional modules associated with ADGRV1 applying affinity proteomics (Knapp et al., 2019; 2022; Kusuluri et al., 2021; Krzysko et al., 2022). Here, we compared the interactomes of ADGRV1 and CIB2 identified by tandem affinity purification (TAP) and found that there was a large overlap in terms of the interacting proteins included. Interestingly, the data sets for both proteins include all eight subunits of the TRiC/CCT chaperonin complex, which is essential for the correct folding of client protein substrates such as actin and tubulin and thereby for the organization of the entire cytoskeleton (Dekker et al., 2008; Brackley and Grantham, 2009). CCT proteins are specifically enriched at the base of primary cilia, suggesting a role in cilia maintenance and/or cell cycle regulation (Seixas et al., 2010; Seo et al., 2010; Kypri et al., 2014). Recently, mutations in the *CCT2* gene have been related to LCA, a severe visual impairment beginning in infancy (Minegishi et al., 2016). Three gene products that are associated with Bardet-Biedl sydrome (BBS) - MKKS/BBS6, BBS10 and BBS12 - have high sequence identity with CCTs (Alvarez-Satta et al., 2017). Together, the BBS-type chaperones and the TRiC/CCT chaperonin complex cooperate in the assembly of the BBSome (Seo et al., 2010; Zhang et al., 2012). The BBSome is a heterooctomeric protein complex consisting of seven BBS proteins: BBS1, BBS2, BBS4, BBS5, BBS7, BBS8, BBS9, and BBIP10 protein (Jin and Nachury, 2009). It plays a key role in primary cilia homeostasis and is essential for the transport of cargo vesicles to primary cilia and the intraflagellar transport (IFT) of membrane cargo within the ciliary shaft (Jin et al., 2010).

Here, we show that ADGRV1 and CIB2 are not only associated with the TRiC/CCT chaperonin complex but also bind to the three BBS chaperones. Moreover, we demonstrate that both USH proteins mutually interact and partially co-localize with the TRiC/CCT subunit CCT3 in the ciliary region of photoreceptor cells. We further demonstrate that the chaperonin complex is essential for the ciliary import of ADGRV1. Our data indicate a functional relation between protein networks involved in the pathomechanisms underlying USH, BBS and LCA.

## Materials and methods

### Constructs and plasmids

For tandem affinity purification (TAP), CIB2 isoform 1 (O75838-1, aa 1-187) was Strep II-FLAG (SF)-tagged at the N terminus. Plasmids used for pulldowns and immunoprecipitation coded for Strep-II-FLAG (SF)-tagged ADGRV1a (Uni-Prot ID Q8WXG9-1, aa 4340-6306), (HA-tagged ADGRV1_CTF (Uni-Prot ID Q8WXG9-1, aa 5891-6306), HA-tagged ADGRV1_ICD (Q8WXG9-1, aa 6155-6306), FLAG-myc-tagged CCT3 (P49368-1) and mRFP-tagged BBS6 (Q9NPJ1-1, aa 2-570), BBS10 (Q8TAM1-1, aa 2-723) and BBS12 (Uni-Prot ID Q6ZW61-1, aa 2-710).

### Cell culture

hTERT-RPE1 cells and HEK293T cells were cultured in Dulbecco’s modified Eagle’s medium (DMEM) containing 10% heat-inactivated fetal calf serum (FCS). Cells were transfected with GeneJuice® (Merck Millipore) according to the manufacturer’s instructions.

### Tandem affinity purification (TAP)

Three TAPs were performed for CIB2 as described (Gloeckner et al., 2007; Knapp et al., 2019). In brief, SF-CIB2 was overexpressed in HEK293T cells for 48 h. Mock-treated cells were used as a control. The cells were lysed, and the lysate was cleared by centrifugation. The supernatant was then subjected to a two-step purification on Strep-Tactin®Superflow® beads (IBA) and anti-FLAG M2 agarose beads (Sigma-Aldrich). Competitive elution was achieved by Desbiothin (IBA) in the first step and FLAG® peptide (Sigma-Aldrich) in the second step. The eluate was precipitated by methanol-chloroform and then subjected to mass spectrometric analysis.

### Mass spectrometry

Mass spectrometry was performed as previously described (Boldt et al., 2016). SF-TAP-purified protein complexes were solubilized before subjecting to trypsin cleavage. Resulting peptides were desalted and purified using stage tips before separation on a Dionex RSLC system. Eluted peptides were ionized by Nano spray ionization and detected by an LTQ Orbitrap Velos mass spectrometer (Thermo Fisher Scientific). Raw spectra were searched against the human SwissProt database using Mascot and the results were verified by Scaffold (Version 4.02.01, Proteome Software Inc.) to validate MS/MS-based peptide and protein identifications. The mass spectrometry proteomics data have been deposited to the ProteomeXchange Consortium via the PRIDE (Perez-Riverol et al., 2022) partner repository with the dataset identifier PXD042629.

### Data processing

Mass spectrometry data of SF-tagged CIB2 were compared to data for mock-transfected cells. Proteins that occurred in the mock dataset were not considered for subsequent analysis of CIB2 data. The identified prey in CIB2-TAPs were compared with the data for ADGRV1a from Knapp et al. (2022). Gene names (according to HGNC) of ADGRV1 and CIB2 prey were used as input for the Cytoscape (http://www.cytoscape.org/) plugins STRING (http://apps.cytoscape.org/apps/stringapp) and ClueGO (Bindea et al., 2009). The parameter *confidence (score) cutout* was set to 0.40 and the parameter *maximum number of interactors* was set to 0 for STRING analysis. ClueGO v2.3.3 was used for Gene Ontology (GO) term enrichment analysis. Network specificity was set to default (medium). Only GO terms that are based on experimental data (setting: All_Experimental (EXP, IDA, IPI, IMP, IGI, IEP)) were included for the enrichment analysis and only pathways with a pV ≤ 0,05 were considered.

### RFP-Trap® analysis

RFP-fused proteins were immobilized on RFP-Trap® agarose beads (ChromoTek) and used for co-precipitation assays according to the manufacturer’s protocol. Briefly, cell lysates from co-transfected HEK293T cells (RFP-tagged proteins or RFP alone together with HA- or SF-tagged proteins, respectively) were suspended in lysis buffer (10 mM Tris-Cl, pH 7.5, 150 mM NaCl, 0.5 mM EDTA, 0.5% NP-40), spun and the supernatant was diluted to 1 mL in dilution buffer (10 mM Tris–Cl, pH 7.5, 150 mM NaCl, 0.5 mM EDTA). Fifty microliters were separated as input (total cell lysate) and samples were added to equilibrated beads for 2 h at 4°C under constant shaking. After washing, precipitated protein complexes were eluted with SDS-sample buffer and subjected to SDS–PAGE and western blotting.

### Immunoprecipitation

For co-IP FLAG-myc-CCT3, and HA-ADGRV1_CTF (HA-ADGRV1_ICD, HA-CIB2, HA-centrin) were expressed in HEK293T cells and lysed in TAP lysis buffer. Co-IP was performed using anti-FLAG M2 beads from Sigma according to the manufacturer’s protocol. Briefly, cell lysates were incubated with anti-FLAG M2 beads for 1 h at 4°C. Reciprocal Co-IPs were performed with anti-HA agarose beads from Biotool. After three washing steps with TAP washing buffer, samples were eluted with SDS-sample buffer and subjected to SDS-page and Western blot, using antibodies against the FLAG- and HA-tag.

### GST pull-down assay

Glutathione S-transferase (GST), GST-tagged BBS6 and 7xHis-tagged ADGRV1-ICD expressed in *E. coli* BL21 AI following the manufacturer’s instructions. Equal amounts of GST or GST fusion protein were mixed with lysates of His-tagged ADGRV1-ICD and a protease inhibitor mix (Sigma-Aldrich). Samples were incubated overnight at 4°C followed by incubation with 50 μL glutathione sepharose beads 4B (Amersham Biosciences) for 45 min with gentle agitation. Beads were centrifugated and washed 4 times with 50 mM Tris-HCl, 150 mM NaCl, 5 mM MgCl2, 1 mM EDTA, 10% glycerol, 0.01% polyoxyethylene-10-lauryl ether, pH 7.5. Subsequently, bound proteins were eluted with SDS sample buffer and subjected to SDS-PAGE and Western blotting.

### Antibodies

The following antibodies were used: mouse anti-CCT3 (Proteintech 60264-1-Ig), mouse anti-CCT2 (Proteintech 68214-1-Ig), mouse anti-CIB2 (Abnova H00010518-A01), rabbit anti-ADGRV1 (Maerker et al., 2008), mouse anti-FLAG M2 **(**Sigma F3165**),** rabbit anti-HA antibody (Roche 11867423001), anti-RFP (Chromotek 5F8), goat-anti-GST (Sigma-Aldrich, SAB 2501414), anti-His antibody (Sigma-Aldrich, SAB1306082), goat anti-centrin 2 antibody (Giessl et al., 2004), anti-paxillin (rabbit polyclonal, Abcam, cat no ab32115; mouse monoclonal, BD Transduction Laboratories, cat no 610052) anti-pericentrin 2 (PCNT2) (Santa Cruz, C-16), mouse anti-Arl13b (Abcam, ab136648). Secondary antibodies conjugated to Alexa 488, Alexa 568, or Alexa 647 were purchased from Molecular Probes (Life Technologies) or from Rockland Inc.

### Animals and tissue dissection

All experiments described herein are conforming to the statement by the Association for Research in Vision and Ophthalmology as to care and use of animals in research. C57BL/6J mice and eGFP-Centrin2 mice (Higginbotham et al., 2004) were maintained under a 12 h light-dark cycle, with food and water *ad libitum*. After sacrificing the animals in CO2 and decapitation, appropriate tissues were dissected. The use of mice in research was approved by District administration Mainz-Bingen, 41a/177-5865-§11 ZVTE, 30.04.2014.

### Immunohistochemistry

The eyes of mice were cryofixed in melting isopentane and cryosectioned as previously described (Wolfrum 1991). Cryosections were placed on poly-L-lysine-precoated coverslips, incubated with 0.01% Tween 20 in PBS, washed several times, covered with blocking solution, and incubated for a minimum of 30 min followed by overnight incubation at 4°C with primary antibodies. Washed cryosections were incubated with secondary antibodies in a blocking solution containing DAPI (1 mg/mL) (Sigma) for 1.5 h at room temperature. After washing, sections were mounted in Mowiol (Roth).

### Immunocytochemistry

hTERT-RPE1 cells were processed for immunohistochemistry as previously described (Krzysko et al., 2022).

### Microscopy

Specimen were analyzed on a Leica DM6000B microscope and 3D deconvoluted with Leica imaging software (three iteration steps). Images were processed with Leica imaging software and Adobe Photoshop CS. Fiji/ImageJ software (NIH, Bethesda) was used for image processing and quantifications.

## Results

### Identification of interacting proteins of CIB2 by tandem affinity purification

To identify novel potential proteins interacting with CIB2 we applied affinity proteomics using tandem affinity purification (TAP) (Boldt et al., 2016). We fused the tandem Strep II-FLAG (SF)-tag to the N-terminus of the CIB2 isoform 1 (Figure 1). The SF-tagged CIB2 was expressed in HEK293T cells, and then subjected to TAP as described previously (Gloeckner et al., 2007; Knapp et al., 2019). Recovered protein complexes were separated by liquid chromatography and the peptide content was determined with tandem mass spectrometry (LC-MS/MS). To identify interacting proteins the raw spectra were searched against SwissProt databases and the results were verified by Scaffold. By applying these strategies we identified 386 potential novel interactors for CIB2 (Supplementary Table S1).

### Comparison of the interactome of CIB2 and ADGRV1 revealed a high degree of overlap

We compared the CIB2 TAP data with the data set which we previously described for ADGRV1a (Knapp et al., 2022). This comparison revealed a high degree of overlap (Figure 1C; Supplementary Table S1), which we did not observe for ADGRV1 and TAP results from other USH proteins (e.g., for SANS (USH1G) and harmonin (USH1C), not shown). We found 270 identical prey proteins in the data sets of CIB2 and ADGRV1 (Figure 1C). Analysis by the Cytoscape plugin STRING revealed that of the 270 shared binding partners of ADGRV1 and CIB2, 244 are highly interconnected (https://string-db.org/, confidence view) and form a common protein network (Figure 1D).

### ADGRV1 and CIB2 physically interact

**FIGURE 2:**
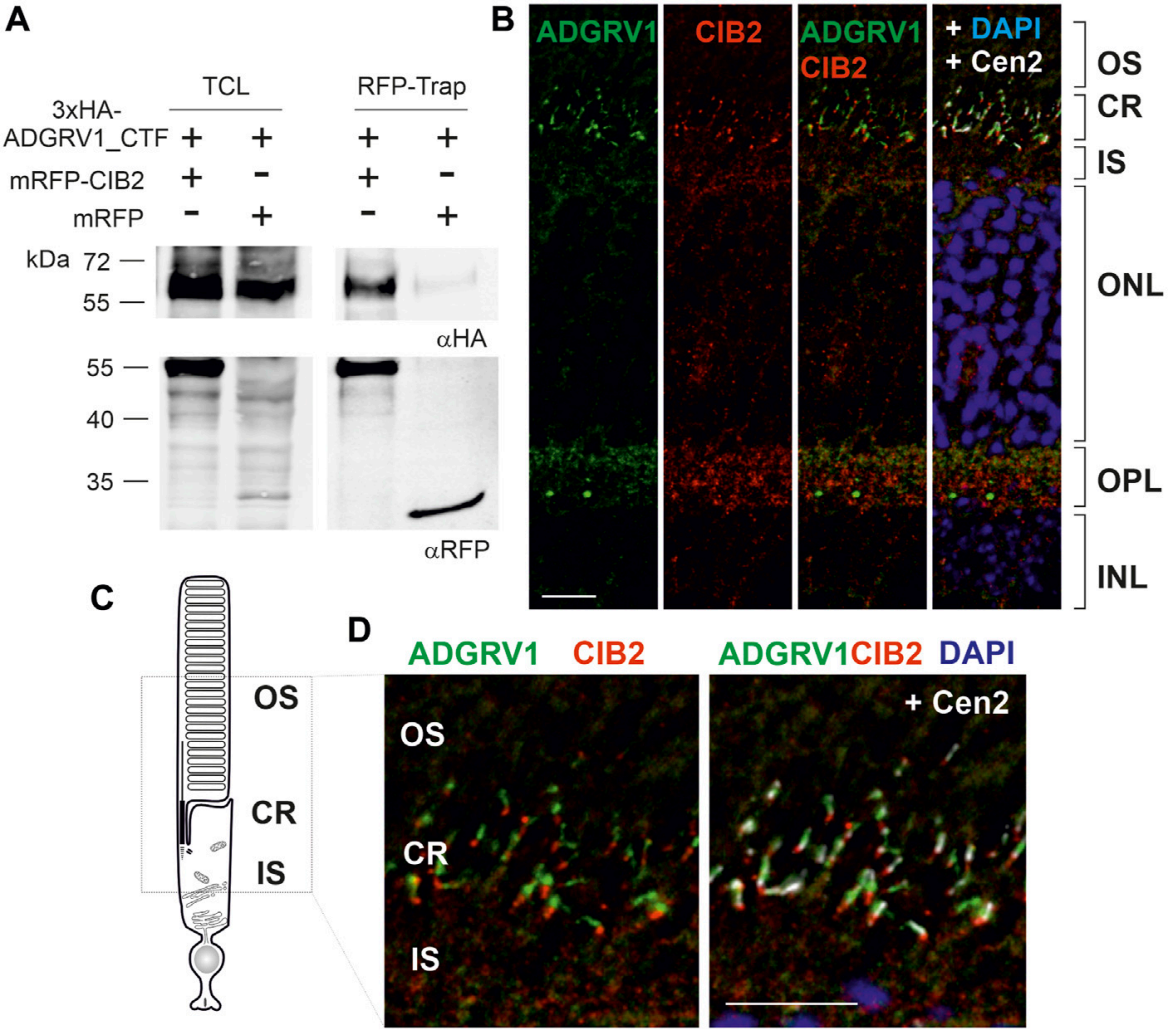
ADGRV1 and CIB2 interact and localize to the ciliary region of photoreceptor cells. **(A)** ADGRV1_CTF is pulled down by RFP-CIB2, but not RFP, in an RFP-Trap^®^. **(B)** Indirect immunolabeling of CIB2, ADGRV1, and the ciliary marker centrin 2 in a cryosection of a murine retina counterstained with the nuclear DNA marker DAPI. Merged images demonstrate ADGRV1 and CIB2 are mainly localized in the ciliary region (CR) and the outer plexiform layer (OPL) where the synapses of photoreceptor cells are present. **(C)** Cartoon of a rod photoreceptor cell: the photoreceptor inner segment (IS) is connected with the outer segment (OS) by the connecting cilium of the CR. **(D)** CIB2 and ADGRV1 localize in close proximity at the proximal end of the basal body, as revealed by the marker protein centrin 2, which localizes to the connecting cilium, the basal body and the adjacent centriole. TCL, total cell lysate; OS, outer segment; IS, inner segment; ONL, outer nuclear layer; OPL, outer plexiform layer; INL, inner nuclear layer; Scale bars: 2b and d = 10 µm.

The high overlap of the ADGRV1 and CIB2 TAP interactome prompted us to test whether both proteins physically interact. To address this question, we performed RFP-Trap® pulldown experiments (Figure 2A). For this we co-expressed the 3xHA-tagged C-terminal fragment of ADGRV1 (3xHA-ADGRV1_CTF) with either RFP-tagged CIB2 (mRFP-CIB2) or RFP alone in HEK293T cells. Cell lysates were then incubated with RFP-Trap® beads to immobilize RFP-CIB2 and RFP, respectively. Subsequent Western blot analysis of the recovered proteins revealed that 3xHA-ADGRV1_CTF was pulled down by RFP-CIB2, but not by RFP alone. We next performed reciprocal immunoprecipitations with anti-HA agarose beads to immobilize 3xHA-ADGRV1_CTF and the appropriate controls (Supplementary Figure S1). In this case, Western blot analysis of the recovered proteins revealed that RFP-CIB2, but not RFP alone, co-immunoprecipitated with 3xHA-ADGRV1_CTF, but not with the HA beads alone. Taken together, these findings demonstrated the interaction between ADGRV1 and CIB2.

### ADGRV1 and CIB2 localize in the ciliary region of photoreceptor cells

Next, we used indirect immunofluorescence to examine whether ADGRV1 and CIB2 are co-distributed in the mouse retina. Immunostaining of both proteins in longitudinal cryosections through the murine retinas showed that ADGRV1 and CIB2 were most prominently localized in the synaptic and ciliary region of photoreceptor cells (Figure 2B). Triple-labeling with antibodies against centrin 2, a marker for the connecting cilium, the basal body and the adjacent daughter centriole of photoreceptor cells (Trojan et al., 2008), further highlighted the ciliary association of CIB2 and ADGRV1 in photoreceptor cells (Figures 2C, D). Immunostaining of pericentrin and Arl13b, common markers for the cilia base and shaft/axoneme of primary cilia, respectively (Mühlhans et al., 2011), as well as CIB2 and ADGRV1 confirmed the localization of CIB2 and ADGRV1 at the cilia base of primary cilia in hTERT-RPE1 cells (Supplementary Figure S2). Co-staining of CIB2 and paxillin, a focal adhesion component, in hTERT-RPE1 cells did not show CIB2 staining in focal adhesions (Supplementary Figure S2A), where ADGRV1 is also localized (Supplementary Figure S2B) (Kusuluri et al., 2021; Güler et al., 2023).

### TAP analysis reveals novel protein complexes associated with ADGRV1 and CIB2

The physical interaction and co-localization of ADGRV1 with CIB2, and the high degree of overlap of their interactomes, indicate their functional relation. To investigate the context of these connections, we further analyzed the shared ADGRV1 and CIB2 network. For this purpose, we used the Cytoscape plugin ClueGO (accessed 10 September 2017) and STRING data (https://string-db.org/) (accessed 20 March 2022) (Bindea et al., 2009), which allows protein enrichment analysis based on Gene Ontology (GO) terms that indicate cellular pathways and processes.

**FIGURE 3:**
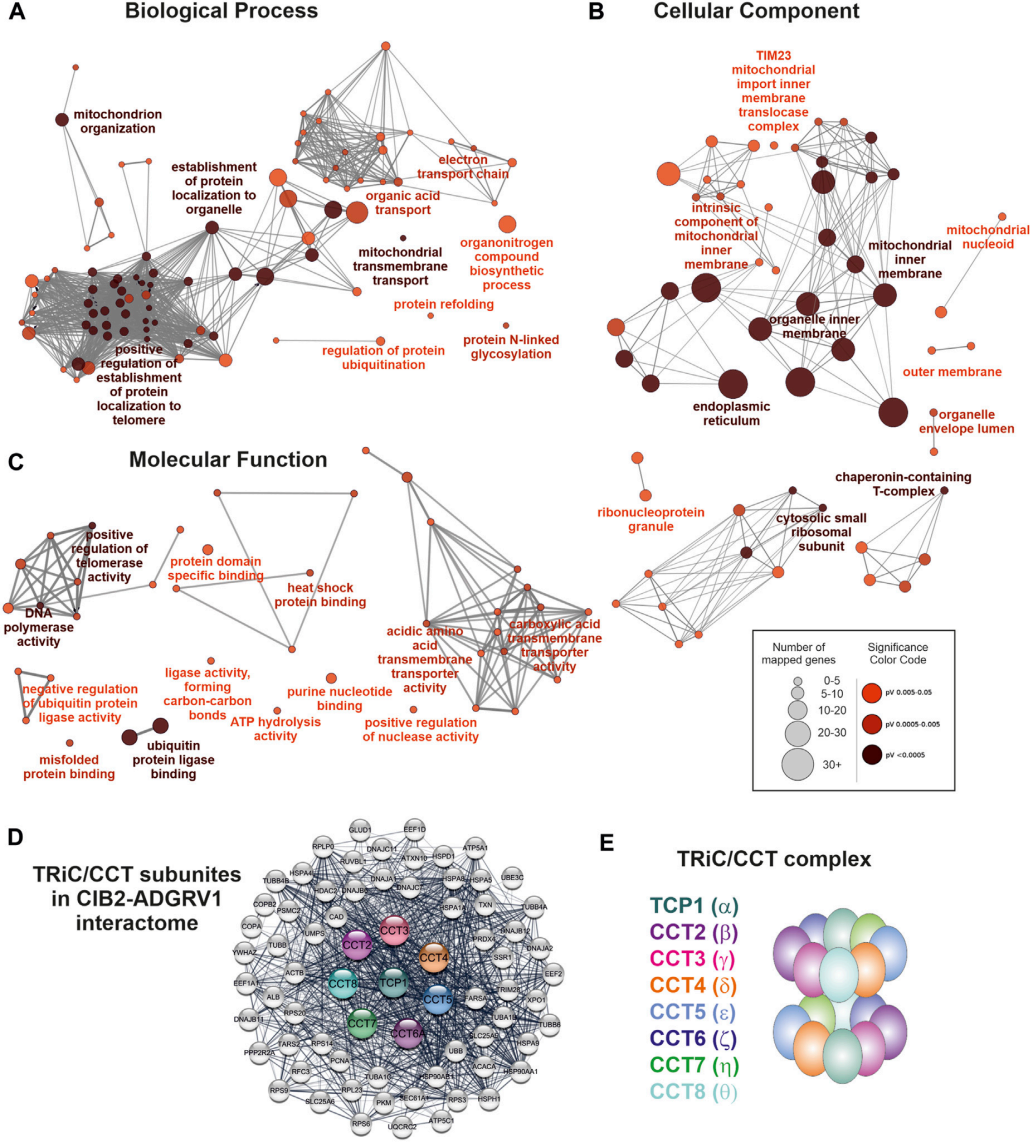
Components of the TRiC/CCT-complex are associated with enriched GO terms and build a subnetwork within the ADGRV1-CIB2 interactome. **(A)** GO term enrichment analysis in the category Biological Process. Depicted are the most significant terms for seven different groups. **(B)** GO term enrichment analysis in the category Cellular Component. Depicted are the most significant terms, for eight different groups. **(C)** GO term enrichment analysis in the category Molecular Function. Depicted are the most significant terms, for eleven different groups. **(D)** Protein network of the TRiC/CCT complex and direct network partners within the ADGRV1-CIB2 interactome. **(E)** The TRiC/CCT complex consists of eight CCT subunits that form a double ring, building up a cylindrical structure with a central cavity.

We searched within the three categories *Biological Process*, *Cellular Component,* and *Molecular Function*. GO terms based on experimental data and a significance pV ≤ 0.05 were considered (Figure 3; Supplementary Tables S2–S4). In the *Biological Process* category, seven groups showed enriched GO terms with the leading terms *positive regulation of DNA biosynthetic process*, *protein localization to nuclear body*, *establishment of protein localization to organelle*, *acidic amino acid transport*, *substantia nigra development*, *protein refolding* and *mitochondrial membrane organization* (Figure 3A). In the *Cellular Component* category, we identified eight GO term groups with the leading terms *extracellular matrix, chaperone complex*, *chaperonin-containing T-complex*, *mitochondrion*, *mitochondrial nucleoid*, *mitochondrial inner membrane presequence translocase complex*, *organelle envelope lumen* and *endoplasmic reticulum* (Figure 3B). In the *Molecular Function* category, we found eleven GO term groups with the leading terms *protein binding involved in protein folding*, *L-glutamate transmembrane transporter activity*, *cation-transporting ATPase activity*, *RNA binding*, *ubiquitin protein ligase binding*, *NADH dehydrogenase activity*, *heat shock protein binding*, *MHC class II protein complex binding*, *cadherin binding* and *protein domain specific binding* (Figure 3C).

The TAP prey, that was related to most of the GO terms in our enrichment analysis, were the eight components of the chaperonin-containing T (CCT)-complex, also known as the TCP1 ring complex (TRiC) (Ghozlan et al., 2022). In addition, STRING analysis demonstrated that the eight CCT subunits are directly connected to numerous additional prey in the ADGRV1-CIB2 interactome (Figure 3D). The CCT complex subunits form a double ring with inter- and intra-ring contact sites, which build up a cylindrical structure with a central cavity, where polypeptides are inserted and folded (Figure 3E).

### ADGRV1 interacts with the CCT3 subunit of the TriC/CCT chaperonin complex

**FIGURE 4:**
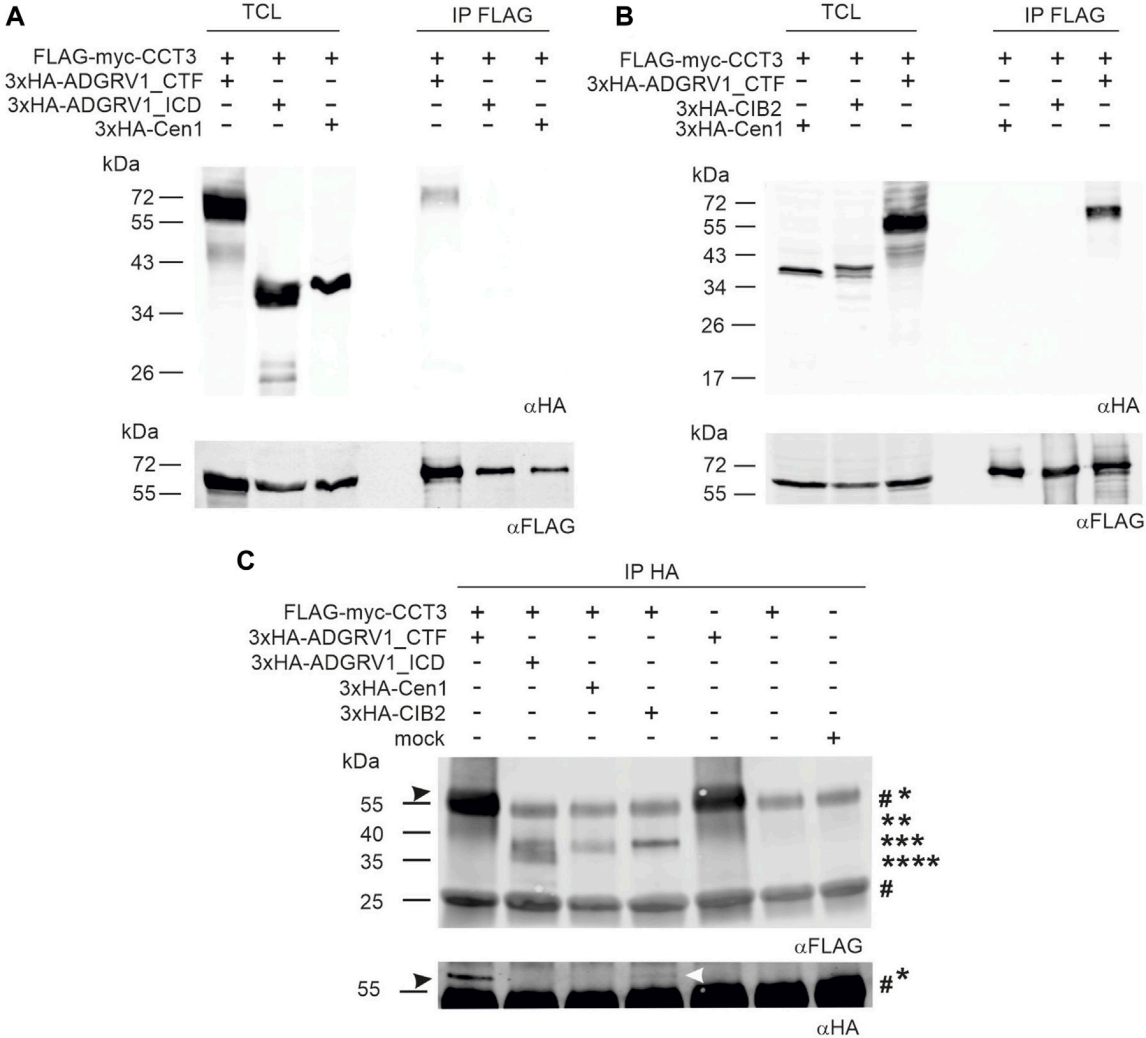
Co-immunoprecipitations of ADGRV1 and CIB2 with CCT3. **(A, B)** anti-FLAG-CCT3-immunoprecipitations: **(A)** Western blot analyses of anti-FLAG-Co-immunoprecipitations from HEK293 cells co-expressing the 3xHA-tagged C-terminal fragment of ADGRV1 (ADGRV1_CTF), the C-terminal intracellular domain (ADGRV1_ICD), or centrin 1 (Cen1), respectively, and the FLAG-myc-tagged CCT3. ADGRV1_CTF but not ADGRV1_ICD nor the negative control Cen1 were recovered, indicating the specific interaction of ADGRV1_CTF with CCT3. **(B)** Western blot analyses of anti-FLAG-Co-immunoprecipitations from HEK293 cells co-expressing 3xHA-tagged ADGRV1_CTF, CIB2, or Cen1, respectively, and the FLAG-myc-tagged CCT3. ADGRV1_CTF but not ADGRV1_ICD nor the negative control Cen1 were recovered, indicating specific interaction of CCT3 with ADGRV1_CTF, but not with CIB2. TCL, total cell lysate; IP, immunoprecipitation. **(C)** Reciprocal anti-HA-ADGRV1/CCT3-immunoprecipitations: Western blot analyses of anti-HA-co-immunoprecipitations from HEK293 cells co-expressing the 3xHA-tagged ADGRV1_CTF, ADGRV1_ICD, CIB2, or Cen1, respectively, and the FLAG-myc-tagged CCT3 or from cells expressing only FLAG-myc-tagged CCT3 as well as mock transfected cells. CCT3 (black arrowhead) was recovered in 3xHA-tagged ADGRV1_CTF but not ADGRV1_ICD nor the negative control Cen1 or “single-expressed” FLAG-myc-tagged CCT3 confirming the specific interaction of ADGRV1_CTF with CCT3 observed in **(A, B)**. In addition, a fade FLAG-myc-tagged CCT3 band was observed in the HA-Co-precipitation with 3xHA-tagged CIB2 (white arrowhead), indicating that CIB2 also interacts with CCT3 in this setting. Molecular weight of the used constructs are indicated: * 3xHA-VLGR1_CTF, ** 3xHA-tagged CIB2, *** 3xHA-Cen1, **** 3xHA-ADGRV1_ICD. The two # indicate the precipitated heavy and light IgG chains of anti-HA, respectively, used for the co-immunoprecipitations (Note: the 3xHA-tagged ADGRV1_CTF band runs very close to the IgG heavy chain.). Loading controls to c are shown in Supplementary Figure S3D.

Since there is increasing evidence for the participation of CCTs in retinal function (Sinha et al., 2014; Minegishi et al., 2016), we further dissected the interaction of ADGRV1 and CIB2 with the complex subunit CCT3. For this, we co-expressed FLAG-myc-tagged CCT3 and 3xHA-ADGRV1_CTF in HEK293T cells and incubated the cell lysate with anti-FLAG® M2 beads. After anti-FLAG pull-downs we subjected the recovered proteins to Western blots and observed binding of CCT3 to ADGRV1_CTF, but not to the intracellular domain of ADGRV1 (ADGRV1_ICD) alone or to the negative control centrin 1, an EF-hand motif-containing Ca^2+^-binding protein (Trojan et al., 2008) (Figure 4A). In contrast, ADGRV1_CTF was not recovered control precipitations with anti-FLAG beads only (Supplementary Figures S3A–C) and CIB2 was not co-precipitated with CCT3 in this experimental setting (Figure 4B).

Next, we performed reciprocal co-immunoprecipitations and incubated the cell lysate with anti-HA beads (Figure 4C). Western blots of anti-HA-pull-downs also showed the binding of CCT3 to ADGRV1_CTF (Figure 4C, right blot, 1st lane) and no binding to the intracellular domain of ADGRV1 (ADGRV1_ICD) or to centrin 1 (Figure 4C), confirming the findings in anti-FLAG-pull-downs (Figures 4A, B). In addition, however, a small substantial portion of CCT3 was precipitated by CIB2 in the anti-HA-immunoprecipitation settings (Figure 4C, fade band indicated by *white arrow*). In contrast, in mock-transfected and single transfected cells expressing FLAG-myc-CCT3, respectively no bands were detectable. Taken together, these findings indicated that CCT3 interacts with the cytoplasmic face of the 7-transmembrane part of ADGRV1, most probably with one of the three intracellular loops and that CIB2 also loosely binds to CCT3.

### CCT3 localizes to the ciliary region of photoreceptor cells

**FIGURE 5:**
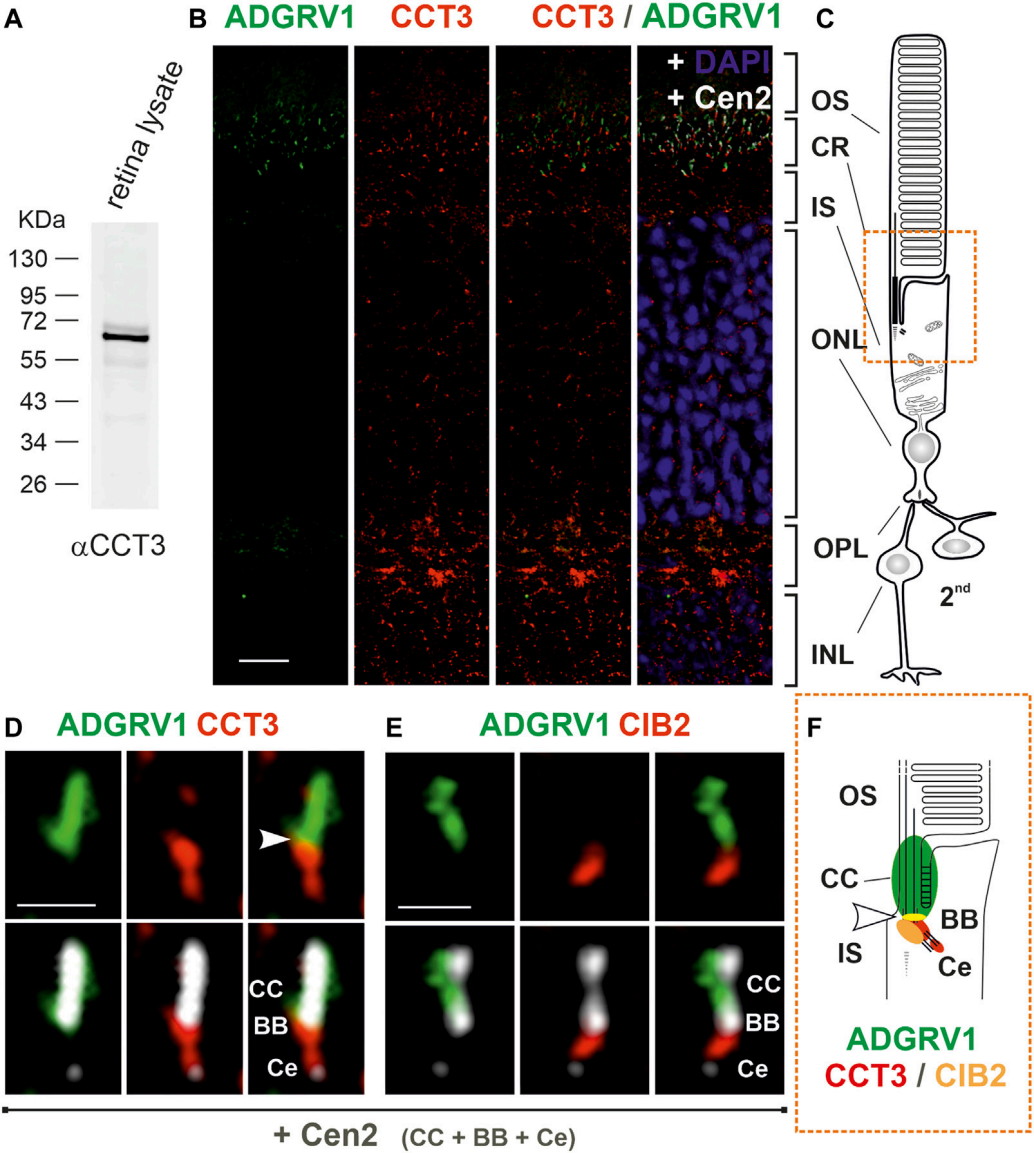
Subcellular localization of CCT3, ADGRV1 and CIB2 in murine retinal photoreceptor cells. **(A)** Anti-CCT3 Western blot of murine retina lysate reveals a band at ∼ 60 kDa, the predicted molecular weight. **(B)** Immunofluorescence triple-labeling of ADGRV1, CCT3 and centrin 2 (Cen2), a marker for the connecting cilium, basal body and centriole, counterstained with DAPI as a nuclear DNA marker on cryosections through a murine retina. **(C)** Schema of a rod photoreceptor cell, linked to 2nd neurons. The comparison of **(B, C)** demonstrates the localization of CCT3 in the outer plexiform layer (OPL, synapse), the outer nuclear layer (ONL, somata), the inner segment (IS) and the ciliary region (CR, orange box) and CCT3—ADGRV1 co-localization in the ciliary. **(D)** Higher magnification of the ciliary region of a CCT3—ADGRV1—Cen2 triple-stained photoreceptor cell reveals the localization of ADGRV1 at the connecting cilium (CC) and CCT3 in the basal body (BB) and the proximal daughter centriole (Ce) with a little overlap (arrowhead). **(E)** Higher magnification of the ciliary region of a CIB2—ADGRV1—Cen2 triple-stained photoreceptor cell from Figure 2D shows the localization of ADGRV1 at the connecting cilium (CC) and CIB 2 in the basal body (BB) with less overlap (arrowhead). **(F)** Schematic representation of the spatial arrangement of ADGRV1, CIB2 and CCT3 in the primary cilium of photoreceptor cells (ciliary region). CIB2 and CCT3 co-localize the basal body proximal to the ADGRV1 present at the connecting cilium (CC). Arrowhead point to the co-localization of ADGRV1 with CCT3 and CIB2. CCT3 is additionally localized in Ce. INL, inner nuclear layer. Scale bars: b = 10 μm; d, e = 1 µm.

It has been previously shown that components of the CCT complex are localized at the base of primary cilia (Seixas et al., 2010; Seo et al., 2010; Kypri et al., 2014). Given that the connecting cilium and photosensitive outer segment of photoreceptor cells represent a modified primary cilium (Roepman and Wolfrum, 2007; May-Simera et al., 2017), we aimed to examine the expression and spatial distribution of CCT3 in the murine retina (Figure 5). In Western blots with antibodies against CCT3 we detected in protein lysate of the murine retina a prominent band at a proximal molecular weight of ∼ 60 kDa, which is in accordance with the predicted size of CCT3 (Figure 5A). Immunohistochemistry in longitudinal cryosections through murine retina cryosections revealed puncta-like staining of CCT3 (Figure 5B). Triple immunostaining of CCT3, ADGRV1, and the ciliary marker protein centrin 2 in retinal sections demonstrated the localization of CCT3 in the outer plexiform layer, the outer nuclear layer, the inner segments of photoreceptors, and indicated co-localization with ADGRV1 in the ciliary region of photoreceptor cells (Figures 5B, C). Higher magnification of the ciliary region of the triple-stained photoreceptor cells confirmed the localization of ADGRV1 at the connecting cilium and the localization of CCT3 in the basal body and proximal daughter centriole with a slight co-localization of both proteins at the junction of the basal body with the connecting cilium (arrowhead) (Figure 5D). A comparison of this staining pattern with the triple staining for ADGRV1, CIB2, and centrin (Figure 5E) indicated the co-localization of CIB2 with CCT3 in the basal body but only a slight co-localization of both proteins with ADGRV1 (Figure 5F). To determine whether the expression profile of CCT3 in the retina can be extended to other molecules of the TRiC/CCT-chaperonin complex, we also examined the expression of CCT2 in the mouse retina (Supplementary Figure S4). Anti-CCT2 Western of retinal lysates revealed bands of the expected size of CCT2 molecules, but also bands of high molecular weight, possibly representing dimers and oligomers (Supplementary Figure S4A), which were previously suspected (Collier et al. (2021). Immunohistochemistry also revealed punctate labeling of CCT2 in the retinal layers (Supplementary Figure S4B), especially in the inner segments and ciliary region of the photoreceptors (Supplementary Figure S4C).

### ADGRV1 and CIB2 interact with the three chaperonin-like BBS proteins

**FIGURE 6:**
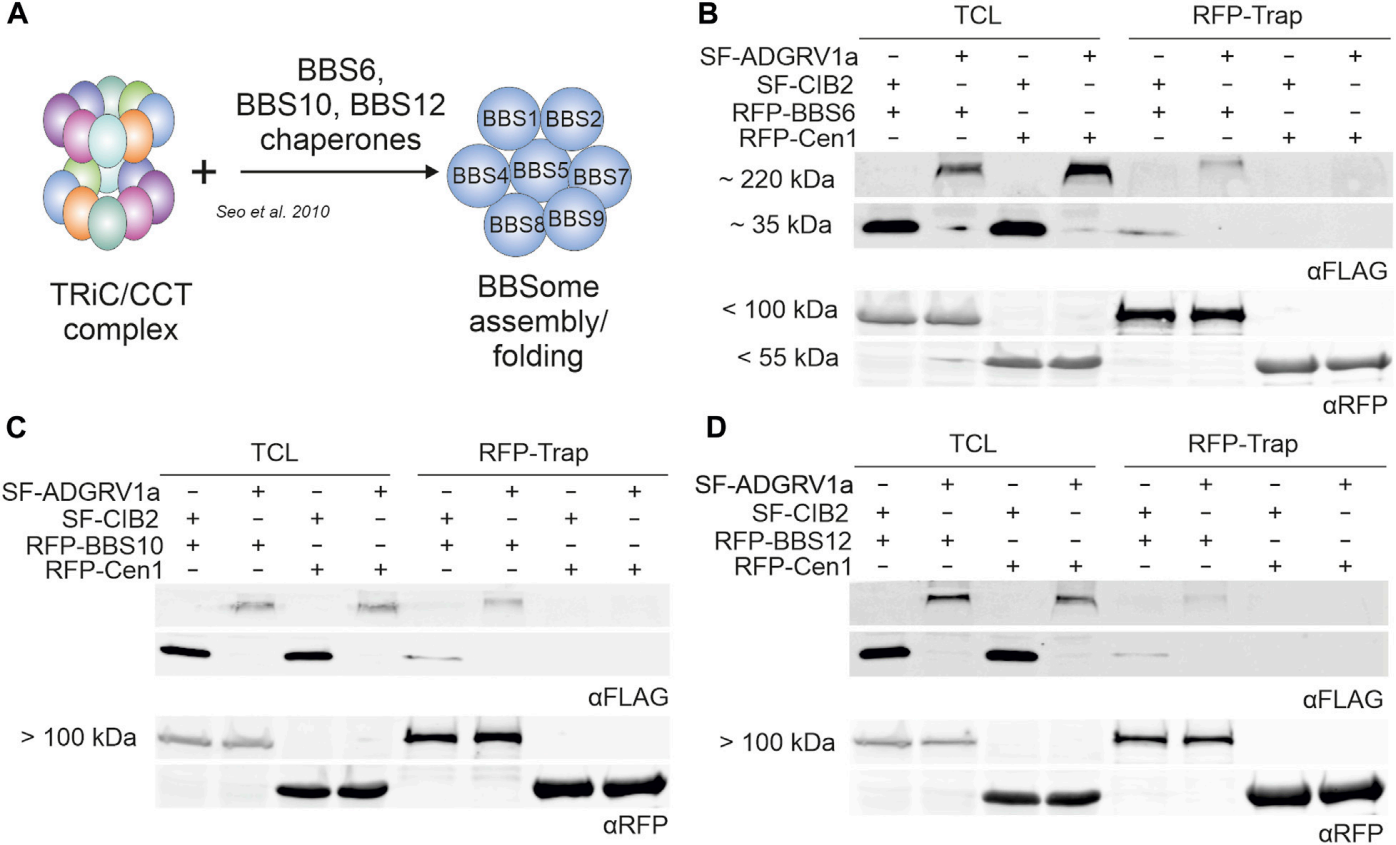
RFP-Trap®-precipitations of ADGRV1 and CIB2 with RFP-tagged chaperonin-like BBS proteins BBS6, BBS10 and BBS12. **(A)** Schematic representation of the assembly of the BBSome mediated by the TRiC/CCT chaperonin ring complex and the chaperonin-like BBS proteins BBS6, BBS10, and BBS12. **(B–D)** Anti-FLAG and anti-RFP Western blots of RFP-Trap®-precipitations from cell lysates co-expressing SF-ADGRV1 or SF-CIB2 together with RFP-tagged BBS6 **(B)**, BBS10 **(C)**, BBS12 **(D)** or RFP-centrin 1 (Cen1), respectively. Both ADGRV1 and CIB2 were recovered by all three RFP-tagged chaperonin-like BBS proteins BBS6, BBS10, and BBS12, but not by the control GFP-Cen1, indicating a specific interaction of ADGRV1 and CIB2 with chaperonin-like BBS proteins. TCL, total cell lysate.

In primary cilia, the interaction of the cytoplasmic TRiC/CCT chaperonin complex with the three chaperonin-like BBS proteins BBS6, BBS10, and BBS12 mediates the assembly of the BBSome (Figure 6A) (Seo et al., 2010). This interaction of CCTs with the chaperonin-like BBS proteins raised the possibility that these may also interact with ADGRV1 and CIB2. To investigate this, we performed RFP-Traps® with ADGRV1a and BBS6, BBS10 and BBS12 (Figures 5B–D). We expressed SF-ADGRV1a or SF-CIB2 together with RFP-tagged BBS6, BBS10, BBS12, or centrin-1, respectively, in HEK293T cells and incubated cell lysates with RFP-Trap® beads for RFP-Trap®-precipitations. Western blot analyses of the recovered proteins revealed the co-precipitation of ADGRV1a and CIB2 with all three BBS chaperone-like proteins (Figures 6B–D). In contrast, neither ADGRV1 nor CIB2 was co-precipitated in the control RFP-Trap®-precipitations with the RFP-tagged centrin 1 (Figures 6B–D). An exemplary GST pull-down with bacterially expressed GST-BBS6 and His-tagged ADGRV1_ICD confirmed the direct interaction between BBS molecules and ADGRV1 (Supplementary Figure S5).

Taken together, the present interaction assays revealed that ADGRV1 and CIB2 interact with the three chaperonin-like BBS proteins.

## Discussion

The knowledge of the function of molecules associated with IRDs is an important prerequisite to define targets for cure and treatment. There is broad agreement in the field that the interacting partners of a protein and the associated protein networks provide clues to cellular modules and thus to the function of a protein (Gavin et al., 2006; Boldt et al., 2016). In our search for interaction partners, we have previously identified numerous putative interacting proteins of the USH2C protein ADGRV1 by applying our affinity capture proteomics strategy (Knapp et al., 2022). The identified molecules pointed to cellular modules in which ADGRV1 acts in concert with those molecules. Recent more detailed studies on some of these modules related to ADGRV1 demonstrated the association of ADGRV1 with focal adhesions where it is involved in mechanosensation during cell motility (Kusuluri et al., 2021; Güler et al., 2023). In addition, we showed the localization of ADGRV1 in mitochondria-associated ER membranes (MAMs), important for the maintenance of Ca^2+^-homeostasis (Krzysko et al., 2022), 2022) and that ADGRV1 controls autophagy processes and cellular proteostasis (Linnert et al., 2023). Moreover, others and we have previously shown that the USH2C protein ADGRV1 is part of the USH interactome interacting with both other USH2 proteins, usherin (USH2A) and whirlin (USH2D), and the two USH1 proteins myosin VIIa (USH1B) and harmonin (USH1C) (Reiners et al., 2005; van Wijk et al., 2006; Michalski et al., 2007; Wang et al., 2023). The putative USH1J protein CIB2 also interacts with the USH proteins whirlin and myosin VIIa (Riazuddin et al., 2012), and the interaction between ADGRV1 and CIB2 that we describe here confirms that CIB2 is part of the USH interactome.

However, the present comparison of the interactomes CIB2 and ADGRV1 revealed that they not only share the USH proteins whirlin and myosin VIIa as binding proteins, but also numerous additional other interaction partners. Of the 386 putative proteins identified as interaction partners of CIB2, 270 (2/3) proteins are also reported to be binding partners of ADGRV1 (Knapp et al., 2019; Knapp et al., 2022) (Figure 1C). Furthermore, present STRING analyses show that most of the common interaction partners of CIB2 and ADGRV1, namely, 244 proteins, are also interconnected in protein networks (Figure 1D) and are part of functional modules, which we confirmed by GO term analyses. We conclude that the functions of CIB2 and ADGRV1 are linked and that both participate in shared processes and joint pathways in the cell.

We have previously described ADGRV1 as a component of focal adhesions interacting with several of their key components such as integrins (Kusuluri et al., 2021; Güler et al., 2023). α/β-integrin heterodimers play essential roles in outside-in and/or inside-out signaling at focal adhesions (Shen et al., 2012; Sun et al., 2016). Although the binding of integrins to CIB2 is well documented and eponymous for CIB2 (Dal Cortivo and Dell’orco, 2022), we could not detect CIB2 at focal adhesions of murine astrocytes (Supplementary Figure S2). The absence of CIB2 from focal adhesions of astrocytes may be due to the fact that CIB2 binds to the cytoplasmic tails of specific α-/β-integrin heterodimers, namely, αIIbβ3 and α7Bβ1 which have been previously found in platelets and megakaryocytes, and in skeletal muscles, but not related to focal adhesions so far (Denofrio et al., 2008; Häger et al., 2008). In any case, according to our data, CIB2 is not expressed in focal adhesions and, consequently, cannot be a functional partner for ADGRV1 there.

Nevertheless, both CIB2 and ADGRV1 are localized at the base of primary cilia, the sensory “ciliary” outer segment of retinal photoreceptor cells and confirmed in the primary cilia model cell line of hTERT-RPE1 cells (Figures 2D, 5E, F; Supplementary Figure S2B). At the ciliary base of photoreceptor cells ADGRV1 has been previously identified as a component of the periciliary membrane complex (PCM) (Maerker et al., 2008; Cosgrove and Zallocchi, 2014; Mathur and Yang, 2015). In the PCM complex, the cytoplasmic domains of ADGRV1 and USH2A are anchored by the scaffold protein whirlin in the cytoplasm of the apical extension of the photoreceptor inner segment where myosin VIIa and USH1G protein SANS are also localized (Liu et al., 1997; van Wijk et al., 2006; Maerker et al., 2008). It is thought that the PCM complex is important for targeting cargos with outer segment destination to the ciliary base and the subsequent handover of these cargos to the ciliary or intraflagellar transport (IFT) systems associated with kinesin 2 or myosin VIIA in the photoreceptor cilium (Maerker et al., 2008; Sedmak and Wolfrum, 2010; May-Simera et al., 2017; Sorusch et al., 2019). The subcellular localization of CIB2 at the ciliary base together with the interaction of CIB2 to several USH proteins of the PMC suggests that CIB2 is also part of the PMC complex in photoreceptor cells.

Besides the PMC the heterooctameric BBSome is localized in the periciliary region of the cilia base. There the BBSome acts as a cargo adapter for membrane proteins such as GPCRs and links cargo to the intraflagellar transport machinery (Jin and Nachury, 2009; Lechtreck et al., 2022). The BBSome is formed via intrinsic protein-protein interactions of BBS proteins of the complex with the BBS-type chaperones BBS6, BBS10 and BBS12 in cooperation with the double ring-shaped TRiC/CCT chaperonin complex (Figures 3E, 6A) (Seo et al., 2010; Zhang et al., 2012). Here, we have demonstrated the interaction of CIB2 and ADGRV1 with both the TRiC/CCT chaperonin complex and the three BBS-type chaperones. Our data also indicate that this interaction occurs at the ciliary base which is in accordance with previous reports on the presence of CCT subunits in ciliary protein networks, which was revealed by TAP (Boldt et al., 2016) and other affinity proteomic screens with centrosomal and ciliary proteins (Sang et al., 2011; Gupta et al., 2015). This interaction of CIB2 and ADGRV1 with the TRiC/CCT-BBS chaperonins indicates molecular links of CIB2 and ADGRV1 to the assembly machinery of the BBSome at the base of primary cilia. Furthermore, these data suggest that CIB2 and ADGRV1 take part in chaperonin functions or alternatively both proteins may represent substrates for the chaperonin complex. Interestingly, there is growing evidence that USH protein complexes are preassembled in the ER (Blanco-Sánchez et al., 2014) before being transported to their final ciliary destination. It is conceivable that they are transported in a pre-folded inactive state and only achieve full functionality by TRiC/CCT-BBS-chaperonin-mediated folding when they reach the ciliary base. However, in the lack of mechanistic insights, the question of whether CIB2 and ADGRV1 are clients of the TRiC/CCT/BBS chaperone complex like the BBSome or contribute to the activity of the chaperone complex will be the subject of further investigations. This may also shed light on the possible interplay between the machineries of the BBSome and the PCM in ciliary transport.

Mutations in *CIB2* and *ADGRV1* were described as causative for human USH, for the subtypes USH1J and USH2C, respectively (Weston et al., 2004; Riazuddin et al., 2012). The association between mutations in *CIB2* with the retinal and vestibular phenotypes in USH1 has recently been debated (Dal Cortivo and Dell’orco, 2022; Delmaghani and El-Amraoui, 2022). However, the close relation by physical interaction, the ciliary co-localization, and the shared protein interactions in a common large interactome found in the present study support a functional interplay of CIB2 and ADGRV1 in retinal photoreceptor cells. Whether this qualifies CIB2 as a USH1 gene must be determined by future studies.

Are the molecular and functional connections between USH proteins, the components of the TRiC/CCT chaperone complex, and various BBS molecules relevant to ocular diseases? Our results here are consistent with the essential chaperone function of CCTs during the biogenesis of photoreceptor cilia in mice (Sinha et al., 2014), which is supported by the identification of mutations in *CCT2* causing retinal degenerations in LCA patients (Minegishi et al., 2016). Moreover, due to the presence of defects in photoreceptor cilia in cell and animal models for USH, evidence has been accumulating in recent years that USH is considered a retinal ciliopathy (Bujakowska et al., 2017; May-Simera et al., 2017; Grotz et al., 2022) which is consistent with the ciliary association of CIB2 and ADGRV1 described here. In addition, the interaction of both USH proteins with BBS molecules as well as their subcellular localization in photoreceptors may hint at a yet enigmatic function of both proteins at the periciliary membrane complex, which controls transport selectivity of proteins from the inner to the outer segment of photoreceptors via ciliary transport. We have recently observed another molecular link between USH and BBS based on the molecular interaction of the USH1G protein SANS with CEP290 (Sorusch et al., 2014). Like in most BBS genes mutations in *CEP290,* also known as *BBS14*, can lead to other, mostly more severe ciliopathies, such as nephronophthisis (NPHP), Meckel-Gruber syndrome (MKS), and Joubert syndrome (JBTS) but also to non-syndromic retinal dystrophies, namely, LCA (Forsythe and Beales, 2013; McConnachie et al., 2021; Delvallée and Dollfus, 2023). The common visual ciliary phenotype in USH, BBS, and LCA based on the diverse disease molecules participating in common pathways are most probably based on defects in ciliary modules such as post-translational modification by chaperonin complexes.

## Conclusion

The association of CIB2 and ADGRV1 with a larger ciliary network shared by USH, BBS, and certain forms of LCA, strongly suggests a role of both proteins in ciliary cargo selection and transport. This is further supported by the fact that mutations in these proteins affect both rod and cone photoreceptors. The overlapping protein networks of both syndromic retinal dystrophies BBS and USH suggest shared pathomechanisms for both syndromes on the molecular level, which bears the chance to identify common therapeutic targets for the correction of the causative defects in a mutation-independent fashion in patients affected by these ciliopathies.

## Data Availability

Full Western blots presented in the study are included in the article/Supplementary Material. The mass spectrometry proteomics data have been deposited to the ProteomeXchange Consortium via the PRIDE partner repository with the dataset identifier PXD042629.
