# Supplementary material for: Usher syndrome proteins ADGRV1 (USH2C) and CIB2 (USH1J) interact and share a common interactome containing TRiC/CCT-BBS chaperonins
Source: Front Cell Dev Biol. 2023 Jun 22;11:1199069. doi: 10.3389/fcell.2023.1199069 (PMC10323441; doi:10.3389/fcell.2023.1199069)
Supplement: Supplementary file 4 [file DataSheet1.PDF]

Supplementary Material

Usher syndrome proteins ADGRV1 (USH2C) and CIB2 (USH1J) interact and share a common interactome containing TRiC/CCT-BBS chaperonins

Joshua Linnert[1§], Barbara Knapp[1§], Baran E. Güler[1], Karsten Boldt[2], Marius Ueffing[2],

Uwe Wolfrum[1]*

[§]These authors contributed to this work and share first authorship

***Correspondence:** Uwe Wolfrum: wolfrum@uni-mainz.de

1. Supplementary Figures

Supplemental Figure S1:

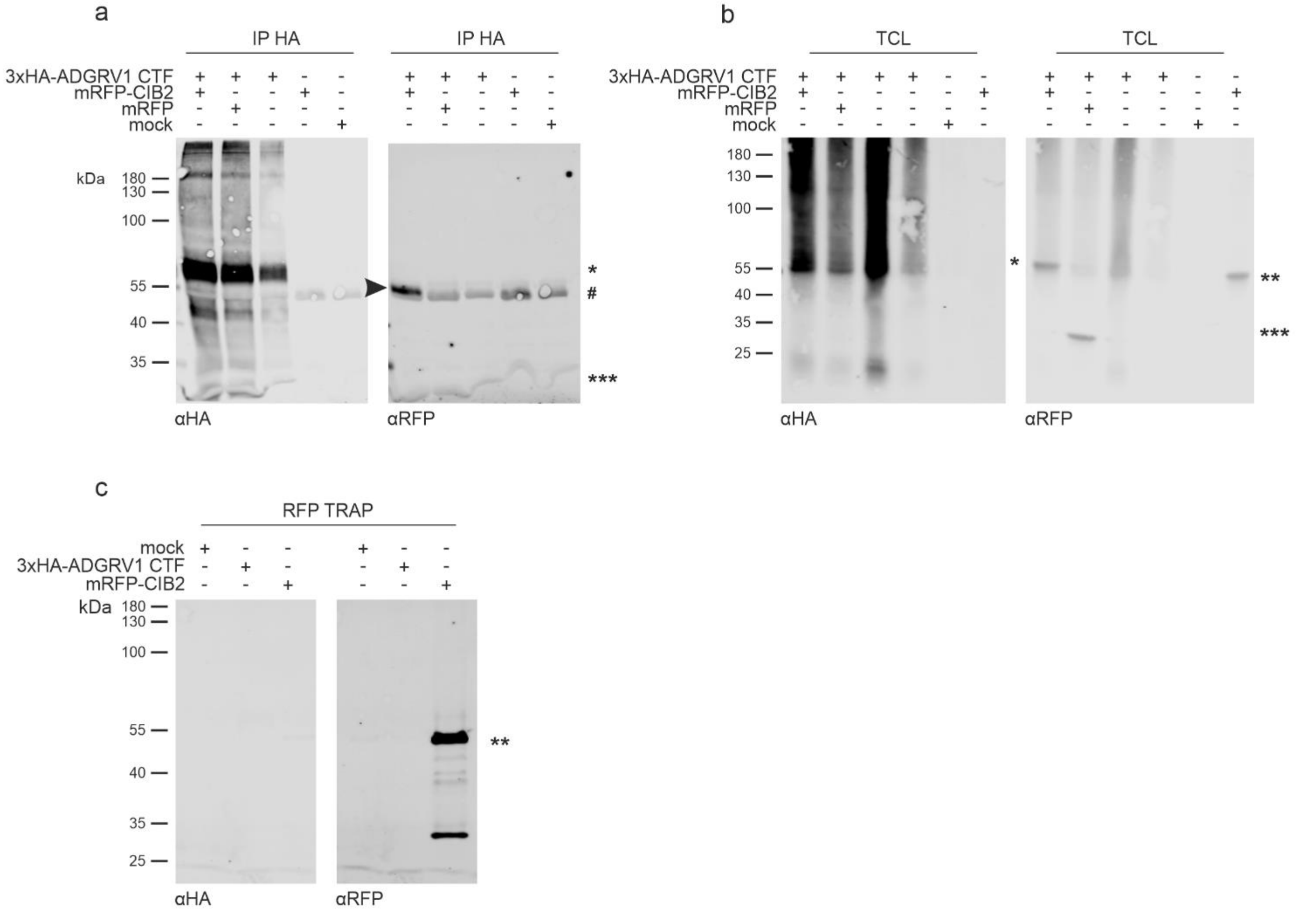

Supplemental Figure S1: **Reciprocal immunoprecipitations with anti-HA agarose beads to immobilize 3xHA-ADGRV1_CTF and the appropriate controls. a)** Reciprocal anti-HA-ADGRV1/CIB2-immunoprecipitations: Western blot analyses of anti-HA-co-immunoprecipitations from HEK293 cells co-expressing 3xHA-tagged ADGRV1_CTF, mRFP-CIB2 or cells expressing only 3xHA-tagged ADGRV1_CTF, mRFP-CIB2, or mock-transfected cells. mRFP-CIB2 (black arrowhead) was recovered from cells co-expressing 3xHA-ADGRV1_CTF but mRFP (*** indicates the molecular weight of mRFP) was not recovered in 3xHA-ADGRV1_CTF expressing cells. Single-expressed mRFP-CIB2 was not immobilized to HA beads, neither was the mock control. Note: the 3xHA-tagged ADGRV1_CTF band runs very close to the IgG heavy chain. These results confirm the specific interaction of ADGRV1 and CIB2 observed in Fig. 2a. **b)** Loading control to a) Total cell lysates (TCL) of HEK293T cells co-expressing the indicated constructs. **c)** Magnetic RFP agarose beads specifically interact with RFP-tagged proteins. RFP agarose beads could only immobilize mRFP-CIB2 not 3xHA-ADGRV1_CTF or the mock control. Molecular weights of constructs used are indicated: * 3xHA-ADGRV1_CTF, ** mRFP-tagged CIB2, *** mRFP. # indicates precipitated heavy IgG chains of anti-HA used for immunoprecipitations.

Supplemental Figure S2:

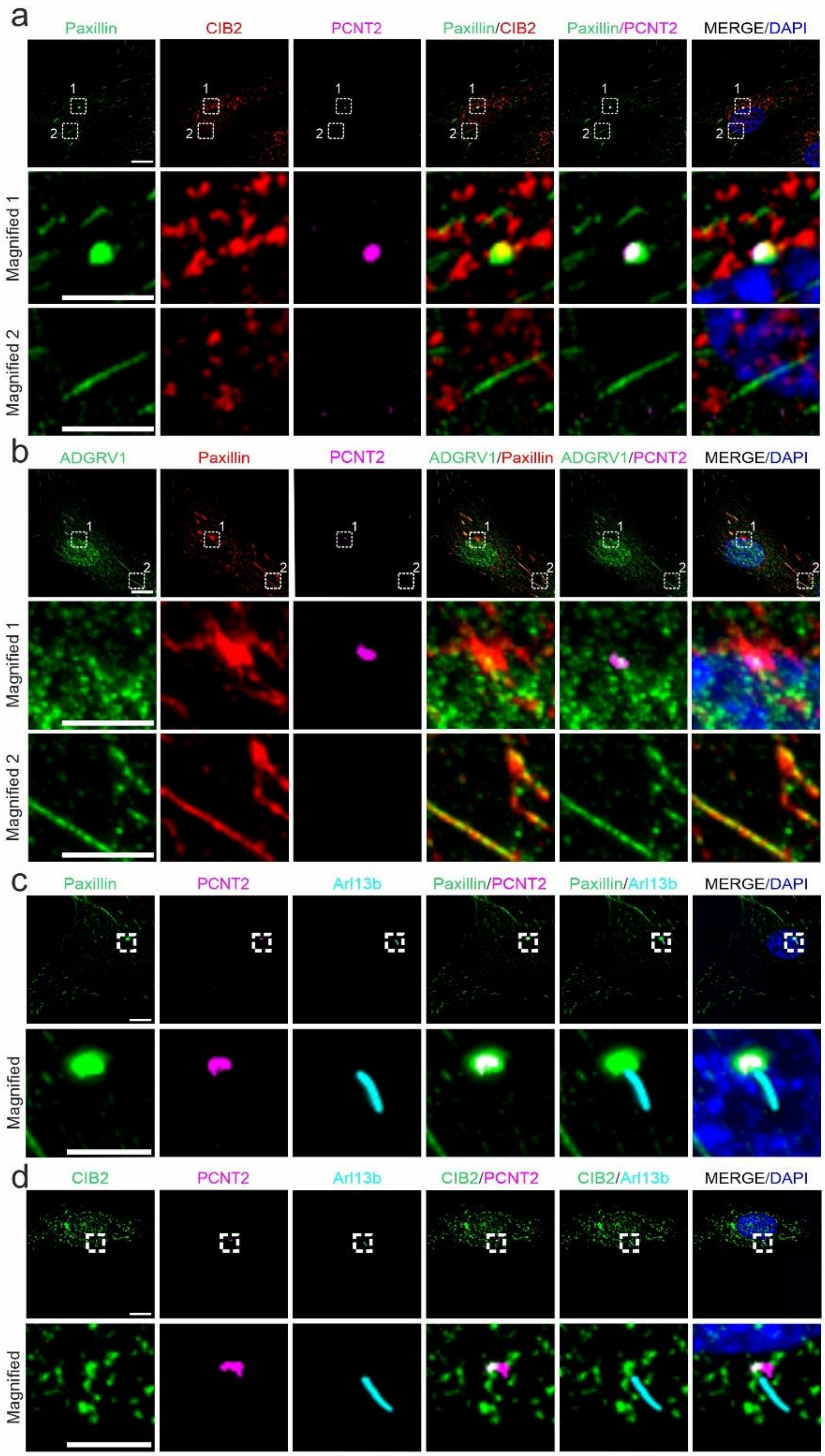

Supplemental Figure S2: **Immunofluorescence analyses of the localization of CIB2 and ADGRV1 in focal adhesions and primary cilia in hTERT-RP1 cells. a)** Immunocytochemical triple-labeling of CIB2, pericentrin (PCTN2), a marker for the periciliary region at the base of primary cilia, and paxilin (ab from rabbit), a characteristic component of focal adhesions demonstrating CIB2 localization at the base of primary cilia but not in focal adhesions. **b)** Immunocytochemical triple-labeling of ADGRV1, pericentrin (PCTN2), and paxillin (ab from mouse) demonstrating localization of ADGRV1 in both the base of primary cilia and in focal adhesions. **c)** Immunocytochemical triple-labeling of paxillin (ab from rabbit), pericentrin (PCTN2), and Arl13b a marker for the axoneme/ciliary shaft of primary cilia demonstrating the localization of paxillin and pericentrin at the base of primary cilia. **d)** Immunocytochemical triple-labeling of CIB2, pericentrin (PCTN2), and Arl13b confirming the localization of CIB2 at the base of primary cilia. Cells in a) to d) are counterstained for nuclear DNA with DAPI. Scale bars: 10 µm, magnification: 5 µm

Supplemental Figure S3:

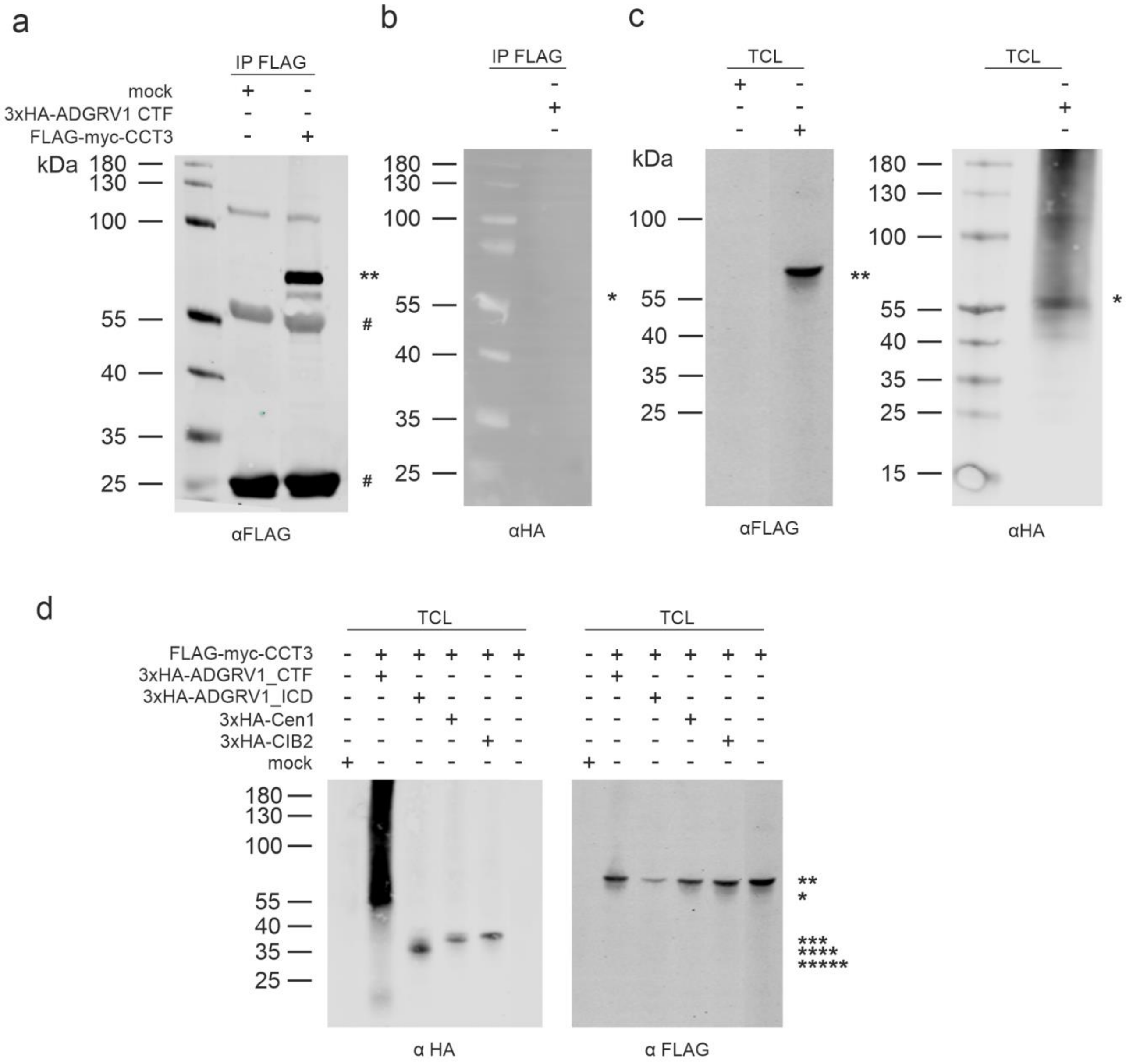

Supplemental Figure S3: anti-FLAG immunoprecipitation controls and loading controls for Figure 4c. a-c) Control for the specificity of anti-FLAG immunoprecipitations (IP FLAG) shown in Fig. 4. **a)** IP FLAG analyzed by anti-FLAG Western blot (left blot) additionally probed with anti-HA (right blot). **b)** IP FLAG analyzed Western blot anti-HA. Only FLAG-myc-tagged CCT3 but not 3xHA-tagged ADGRV1 is precipitated in IP FLAG. **d)** Loading control to Supplemental Figure S3 a-c and Figure 4c. Total cell lysates (TCL) of HEK293T cells co-expressing or single-expressing the indicated constructs. Molecular weights of constructs used are indicated: * 3xHA-ADGRV1_CTF, ** FLAG-myc-tagged CCT3, *** 3xHA-tagged CIB2, **** 3xHA-tagged Cen1, **** 3xHA-ADGRV1_ICD. # indicates precipitated heavy and light IgG chains of anti-HA used for immunoprecipitations.

Supplemental Figure S4:

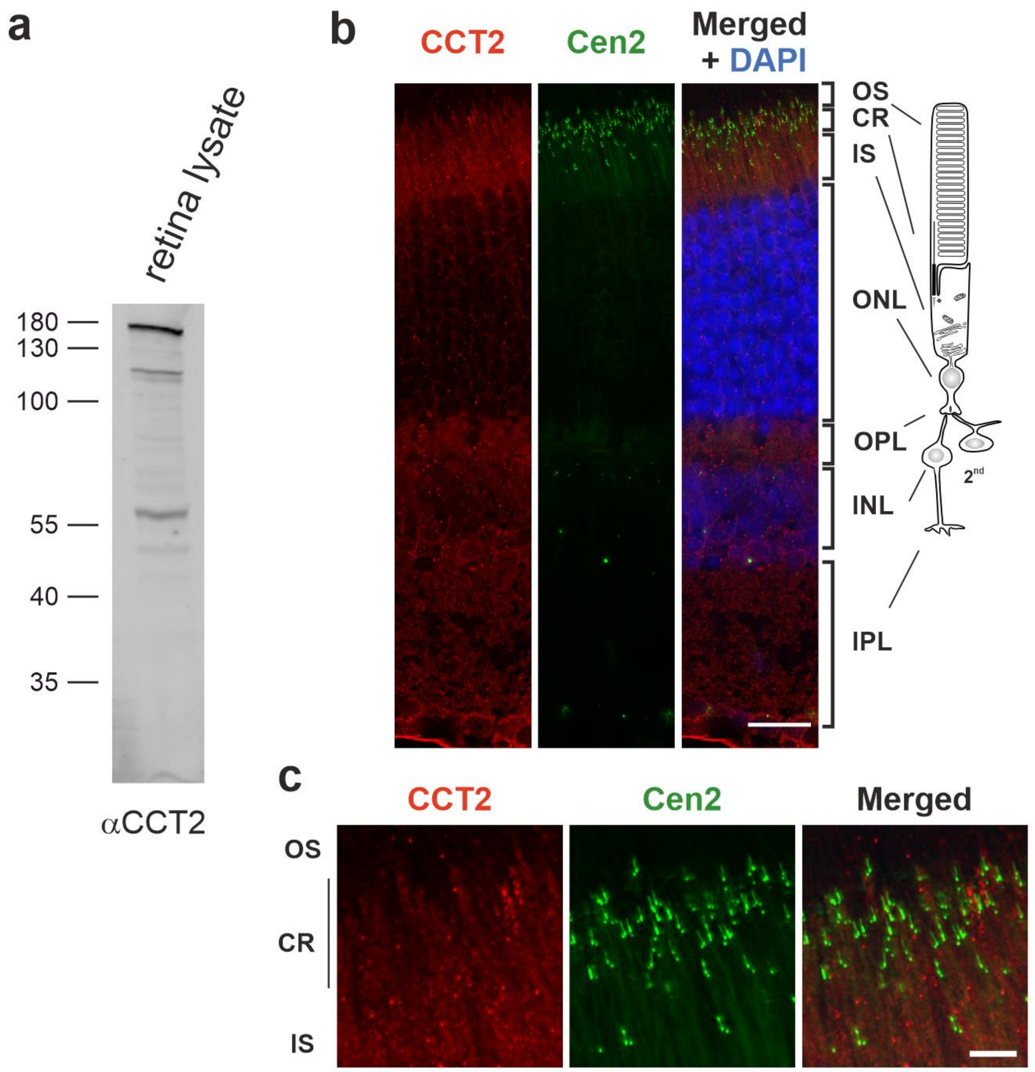

Supplemental Figure S4: Subcellular localization of CCT3, ADGRV1 and CIB2 in murine retinal photoreceptor cells. a) Anti-CCT2 Western blot analysis of murine retina lysate reveals a band slightly above 55 kDa the estimated molecular weight of CCT2 and two higher molecular weight bands which might represent dimers and oligomers of CCT2. Dimer formation has been previously reported by Collier et al. (2001). **b)** Immunofluorescence staining of CCT2 in cryosections through the retina of eGFP-Centrin2 mice (Higginbotham et al. 2004) counterstained with DAPI as a nuclear DNA marker. Centrin 2 (Cen2) represents a marker for the connecting cilium, basal body and centriole. Schema indicates retinal layers related to a rod photoreceptor cell, linked to 2nd neurons. Punctate staining of CCT2 is present in most retinal layers, but most prominent in the ciliary region (CR, eGFP-Centrin2 positive) and the inner segment (IS) of photoreceptor cells. OS = Outer segment; ONL = outer nuclear layer (somata of photoreceptor cells), OPL = outer plexiform layer (photoreceptor synapses to 2nd neurons), INL = inner nuclear layer (somata of 2nd retinal neurons). **c)** Higher magnification of the ciliary region of photoreceptors in eGFP-Centrin2 mouse retina stained for CCT2. Scale bars: b: 10 µm and c: 5 µm.

Supplemental Figure S5:

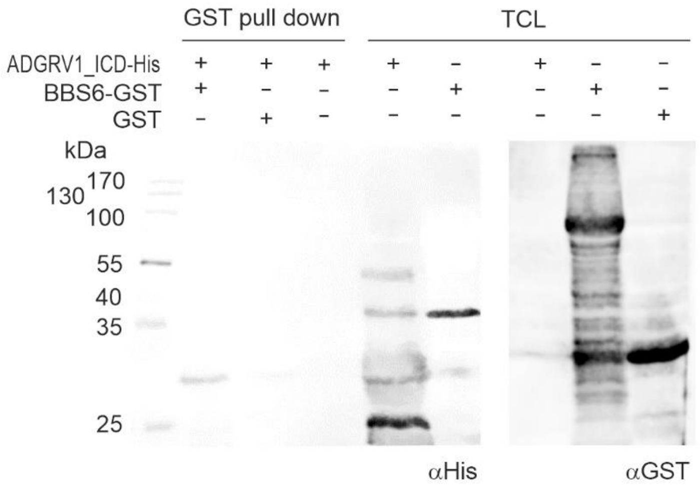

Supplemental Figure S5: Binding of ADGRV1_ICD to BBS6 revealed by a GST pull down. His-tagged ADGRV1_ICD is recovered by GST-BBS6 but not by GST alone. TCL = whole cell lysate. Note: the 40 kDa molecular weight marker is very fade, almost not visible on the blot.

2. Supplementary Tables

Supplemental Tables S1 Overlap of hits from CIB2 and VLGR1a TAPs.xlsx

Supplemental Tables S2 BP only Exp.pV 0.05 ClueGO. xls

Supplemental Tables S3 CC only Exp.pV 0.05 ClueGO.xls

Supplemental Tables S4 MF only Exp.pV 0.05 ClueGO.xls

3. Appendix

The Western blots shown in Figures 2 a, 4 a,b, 5 a, 6 b,c, and d were cropped in the main article for reasons of clarity. Here we provide the uncropped images.

To Figure 2a

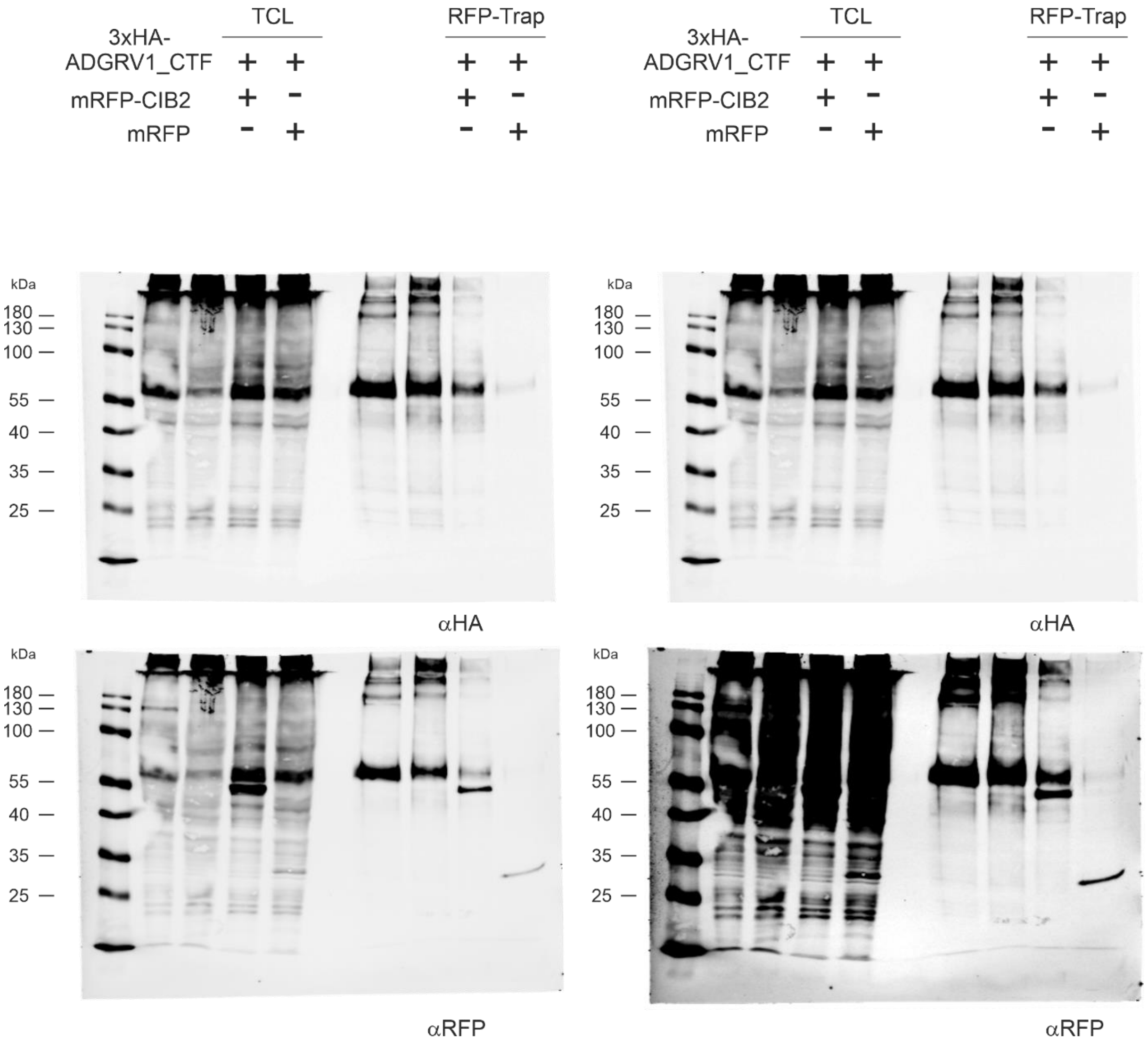

To Figure 4a

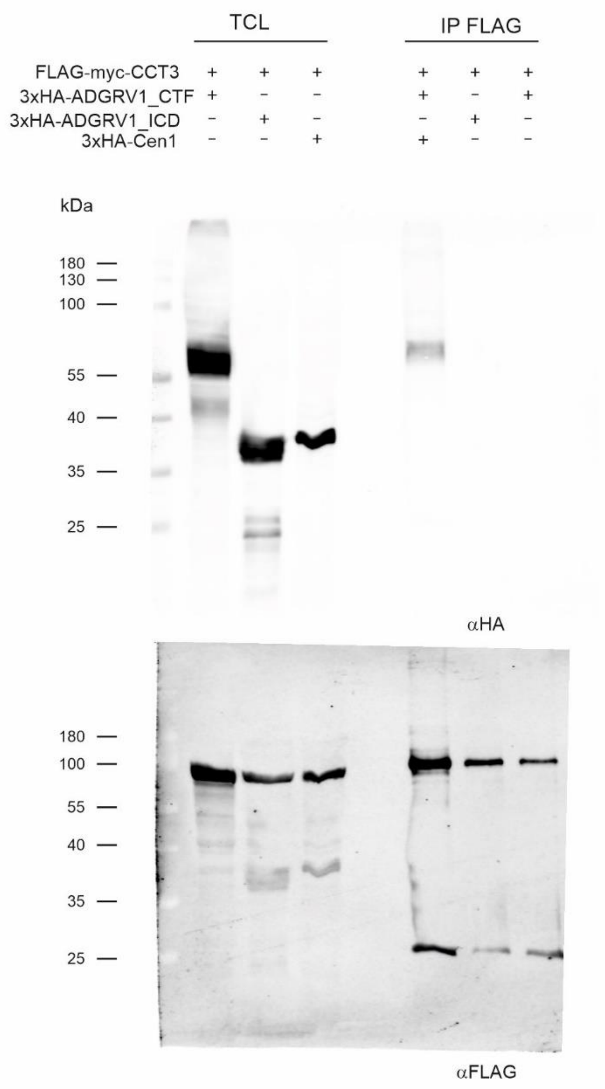

TCL
IP FLAG
FLAG-myc-CCT3 + + + + + +
3xHA-ADGRV1_CTF + − − + − +
3xHA-ADGRV1_ICD − + − − + −
3xHA-Cen1 − − + + − −
kDa
180
130
100
55
40
35
25
αHA
180
100
55
40
35
25
αFLAG

To Figure 4b

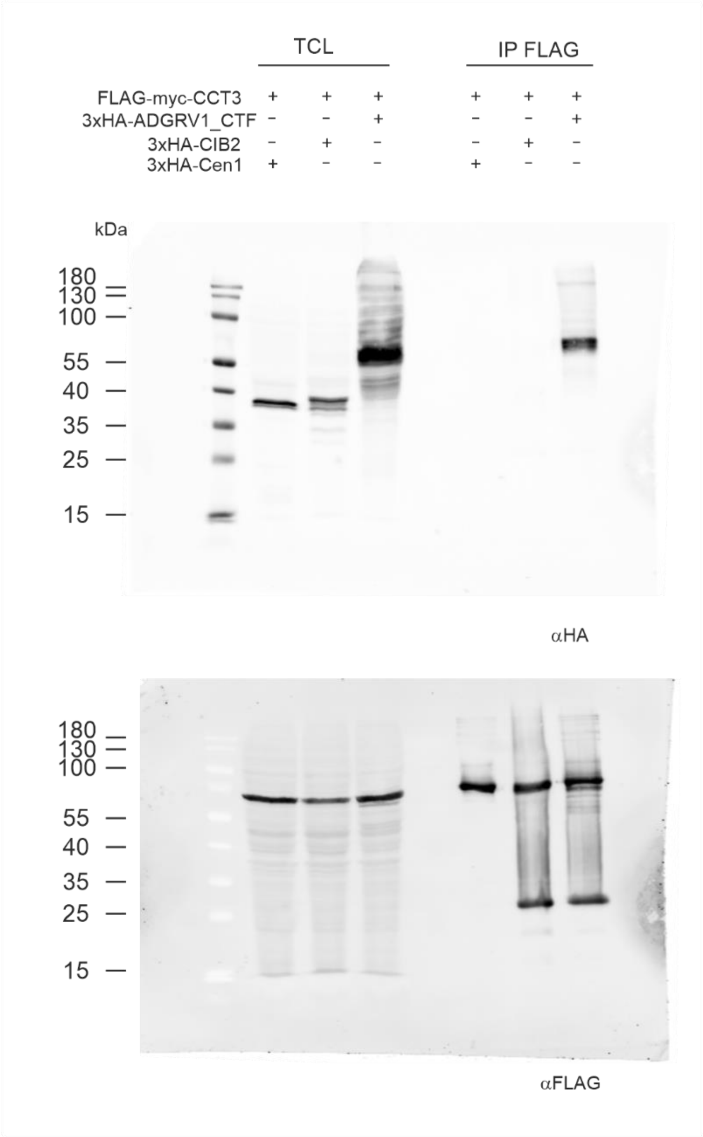
TCL
IP FLAG
FLAG-myc-CCT3 + + + + + +
3xHA-ADGRV1_CTF – – + – – +
3xHA-CIB2 – + – – + –
3xHA-Cen1 + – – + – –
kDa
180
130
100
55
40
35
25
15
αHA
180
130
100
55
40
35
25
15
αFLAG

To Figure 4c

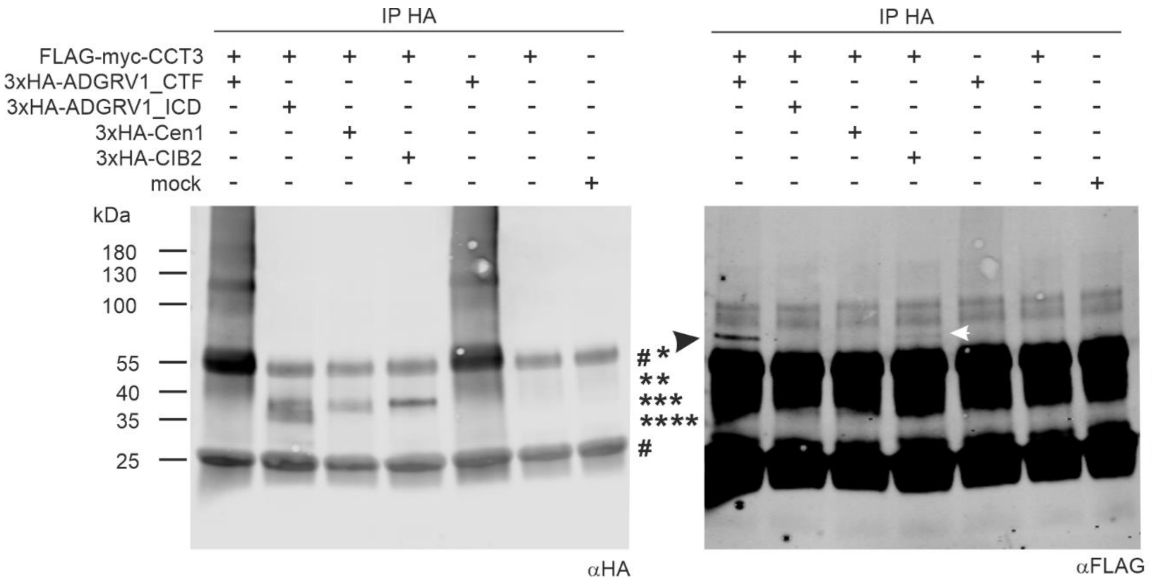

To Figure 5a

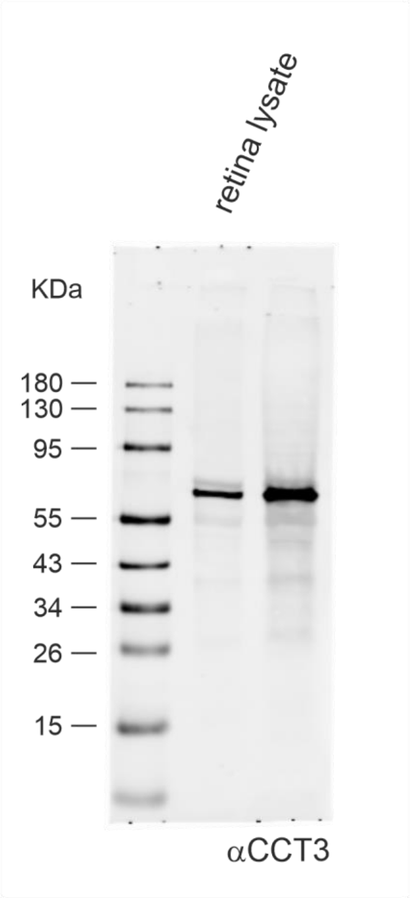

To Figure 6b

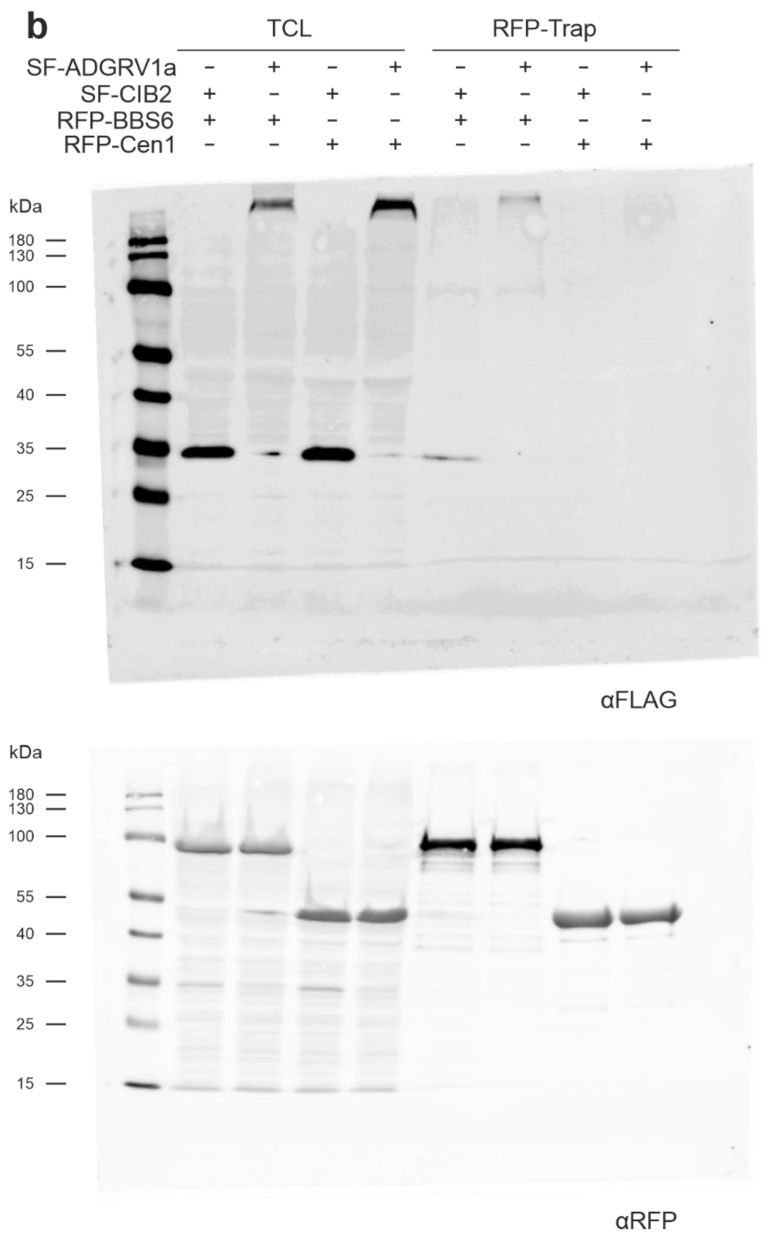
b
TCL
RFP-Trap
SF-ADGRV1a – + – + – + – +
SF-CIB2 + – + – + – + –
RFP-BBS6 + + – – + + – –
RFP-Cen1 – – + + – – + +
kDa
180
130
100
55
40
35
25
15
αFLAG
kDa
180
130
100
55
40
35
25
15
αRFP

To Figure 6c

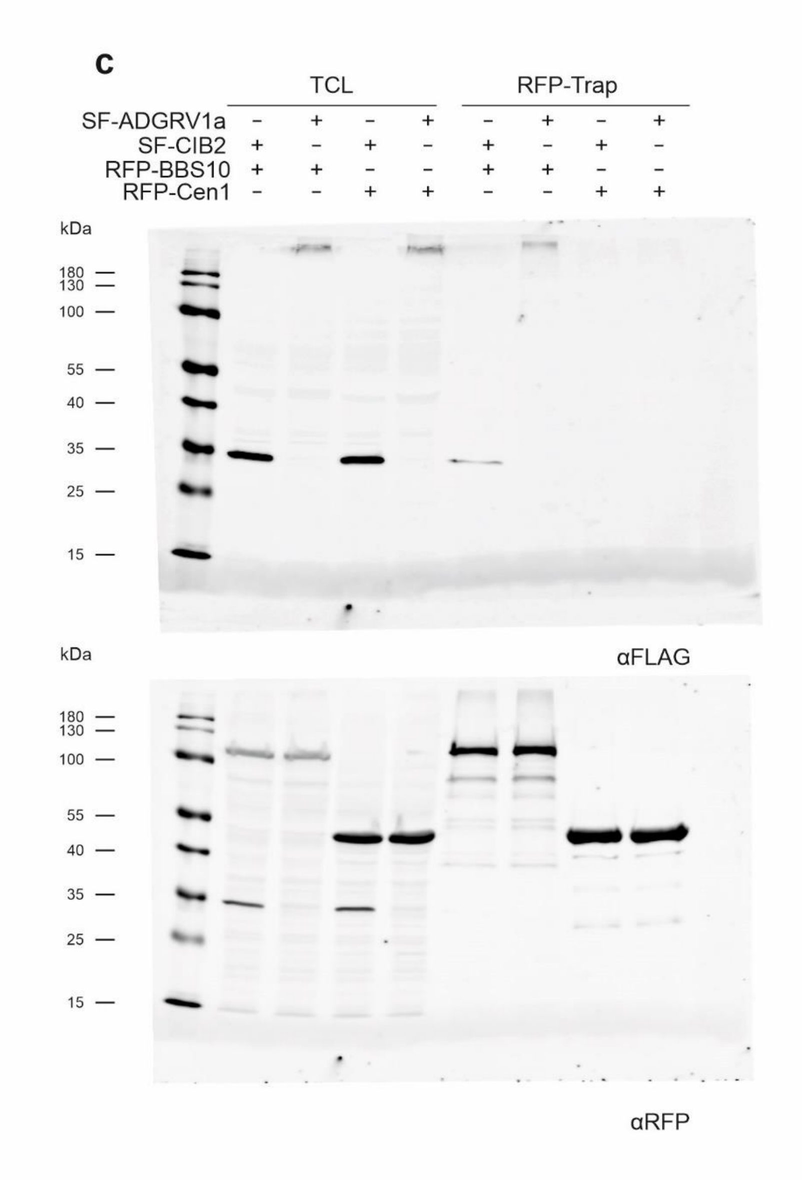
c
TCL
RFP-Trap
SF-ADGRV1a – + – + – + – +
SF-CIB2 + – + – + – + –
RFP-BBS10 + + – – + + – –
RFP-Cen1 – – + + – – + +
kDa
180
130
100
55
40
35
25
15
αFLAG
kDa
180
130
100
55
40
35
25
15
αRFP

To Figure 6d

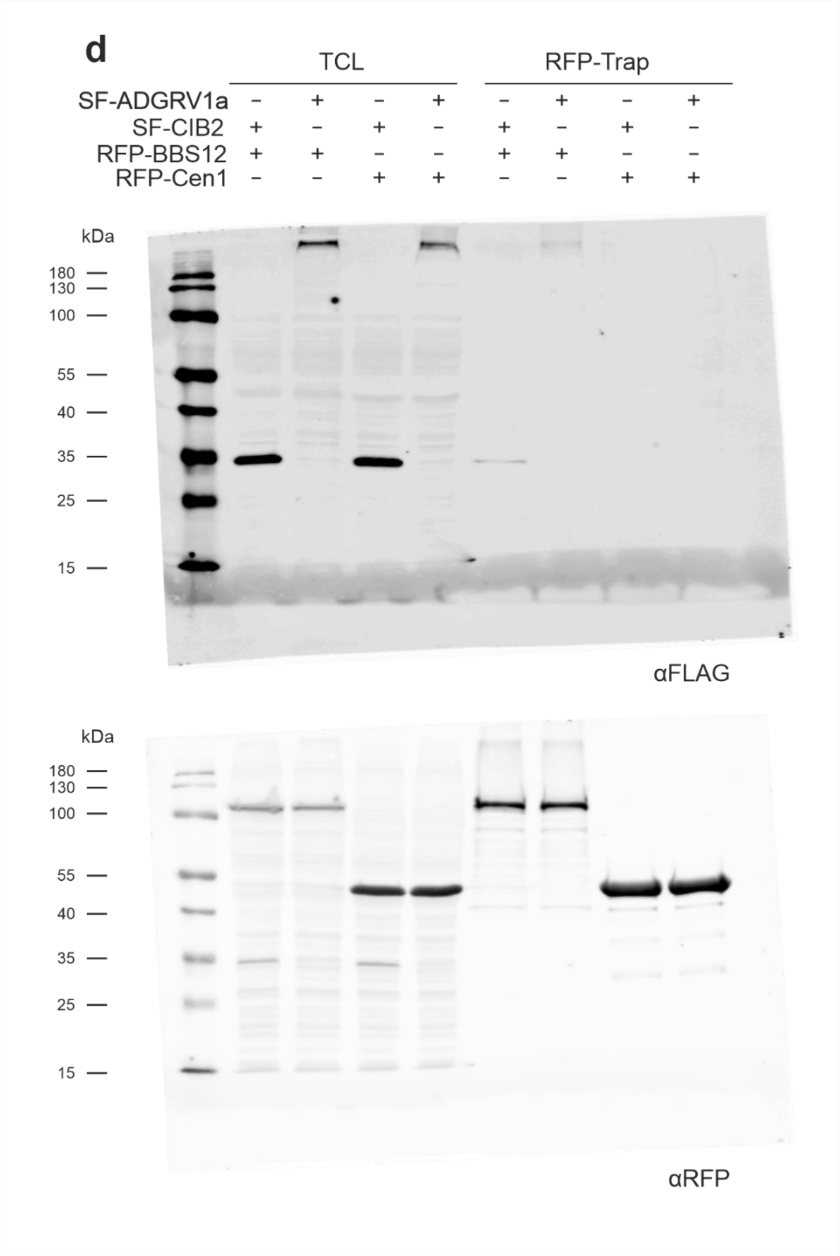
d
TCL
RFP-Trap
SF-ADGRV1a – + – + – + – +
SF-CIB2 + – + – + – + –
RFP-BBS12 + + – – + + – –
RFP-Cen1 – – + + – – + +
kDa
180
130
100
55
40
35
25
15
αFLAG
kDa
180
130
100
55
40
35
25
15
αRFP
